# Ferroptosis: mechanisms and therapeutic targets

**DOI:** 10.1002/mco2.70010

**Published:** 2024-11-20

**Authors:** Qian Zhou, Yu Meng, Jiayuan Le, Yuming Sun, Yating Dian, Lei Yao, Yixiao Xiong, Furong Zeng, Xiang Chen, Guangtong Deng

**Affiliations:** ^1^ Department of Dermatology Xiangya Hospital Central South University Changsha Hunan Province China; ^2^ National Engineering Research Center of Personalized Diagnostic and Therapeutic Technology Changsha Hunan Province China; ^3^ Furong Laboratory Changsha Hunan Province China; ^4^ Hunan Key Laboratory of Skin Cancer and Psoriasis Hunan Engineering Research Center of Skin Health and Disease Xiangya Hospital Central South University Changsha Hunan Province China; ^5^ National Clinical Research Center for Geriatric Disorders Xiangya Hospital Changsha Hunan Province China; ^6^ Department of Plastic and Cosmetic Surgery Xiangya Hospital Central South University Changsha Hunan Province China; ^7^ Department of General Surgery Xiangya Hospital Central South University Changsha Hunan Province China; ^8^ Department of Dermatology Tongji Hospital Huazhong University of Science and Technology Wuhan Hubei China; ^9^ Department of Oncology Xiangya Hospital Central South University Changsha Hunan Province China

**Keywords:** epigenetics, ferroptosis, human disease, lipid peroxidation

## Abstract

Ferroptosis is a nonapoptotic form of cell death characterized by iron‐dependent lipid peroxidation in membrane phospholipids. Since its identification in 2012, extensive research has unveiled its involvement in the pathophysiology of numerous diseases, including cancers, neurodegenerative disorders, organ injuries, infectious diseases, autoimmune conditions, metabolic disorders, and skin diseases. Oxidizable lipids, overload iron, and compromised antioxidant systems are known as critical prerequisites for driving overwhelming lipid peroxidation, ultimately leading to plasma membrane rupture and ferroptotic cell death. However, the precise regulatory networks governing ferroptosis and ferroptosis‐targeted therapy in these diseases remain largely undefined, hindering the development of pharmacological agonists and antagonists. In this review, we first elucidate core mechanisms of ferroptosis and summarize its epigenetic modifications (e.g., histone modifications, DNA methylation, noncoding RNAs, and N6‐methyladenosine modification) and nonepigenetic modifications (e.g., genetic mutations, transcriptional regulation, and posttranslational modifications). We then discuss the association between ferroptosis and disease pathogenesis and explore therapeutic approaches for targeting ferroptosis. We also introduce potential clinical monitoring strategies for ferroptosis. Finally, we put forward several unresolved issues in which progress is needed to better understand ferroptosis. We hope this review will offer promise for the clinical application of ferroptosis‐targeted therapies in the context of human health and disease.

## Graphical abstract

The core molecules driving the induction and execution of ferroptosis represent promising therapeutic targets for treating ferroptosis‐related diseases.

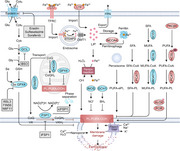

## 1 INTRODUCTION

Regulated cell death (RCD) refers to a controllable and intervenable form of cell death, playing a fundamental role in maintaining homeostasis and biological processes.^1^ Ferroptosis, first described in 2012 by the Stockwell laboratory during a screen for agents selectively lethal to RAS‐mutant cancer cells, represents a unique, nonapoptotic form of RCD.^2^ Unlike apoptosis, autophagy, and necroptosis, ferroptosis is driven by iron‐dependent overwhelming lipid peroxidation of polyunsaturated fatty acids (PUFAs) in membrane phospholipids, underscoring the pivotal role of lipid metabolism in its initiation and execution.^3^ The oxidizable lipids in the form of PUFA‐containing phospholipids (PUFA‐PLs) provide the substrates for ferroptosis execution.^4^, ^5^ Under conditions of iron overload and compromised antioxidant defenses, these lipids undergo uncontrolled peroxidation, leading to plasma membrane permeabilization, rupture, and eventual cell death.^4^, ^5^

The regulation of ferroptosis is complex and involves both epigenetic modulators and nonepigenetic factors such as histone modifications, DNA methylation, noncoding RNAs (ncRNAs) regulation, N6‐methyladenosine (m6A) modification, genetic mutations, transcriptional regulators, and posttranslational modifications (PTMs).^5^, ^6^, ^7^, ^8^ These elements directly or indirectly control the governing mechanisms of ferroptosis, including iron accumulation, lipid metabolism, and antioxidant responses. Dysregulation of the ferroptotic network has been increasingly implicated in the pathogenesis of various diseases, such as cancers, neurodegenerative disorders, organ injuries, infectious diseases, autoimmune conditions, metabolic disorders, and skin diseases.^9^, ^10^ A detailed understanding of ferroptosis and its role in disease pathophysiology paves the way for innovative therapeutic strategies.

To date, several ferroptosis‐targeted therapies have been developed, focusing on modulating the key drivers of ferroptosis and offering potential new treatment options. This review overviews the core mechanisms of ferroptosis, focusing on its prerequisites and execution, and the intricate regulatory network of ferroptosis modulated by epigenetic and nonepigenetic factors was also illustrated. Additionally, we discuss the emerging role of ferroptosis in human disease and summarize the primary pharmacological strategies aimed at modulating this process. Last, we highlight potential clinical monitoring tools for ferroptosis, providing a foundation for future clinical applications.

## 2 CORE MECHANISMS OF FERROPTOSIS

**FIGURE 1:**
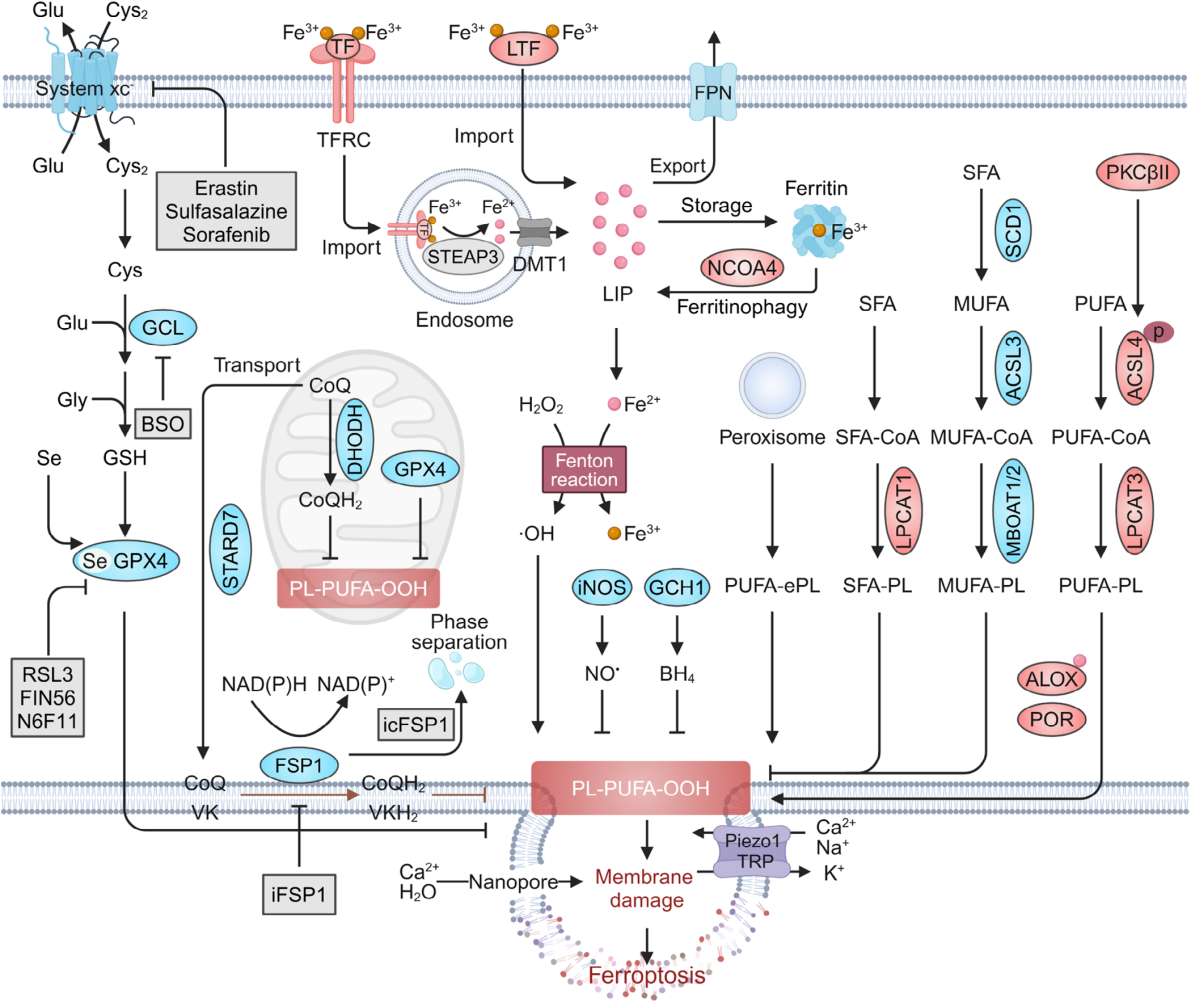
Core mechanisms of ferroptosis. Oxidizable lipids in the cellular membrane, particularly PUFA‐PLs mediated by ACSL4 and LPCAT3, are preferential substrates of iron‐dependent nonenzymatic and enzymatic lipid peroxidation. When GPX4‐dependent or ‐independent antioxidant systems (e.g., FSP1, DHODH, GCH1, iNOS, 7‐DHC) are compromised, cellular defense against lipid peroxidation diminishes, allowing uncontrolled lipid peroxidation. The lethal accumulation of lipid peroxidation overwhelms antioxidant defenses and membrane repair capacity, activating mechanosensitive cation channels, disrupting ion homeostasis, and ultimately leading to membrane rupture and ferroptotic cell death. ACSL4, acyl‐CoA synthetase long‐chain family member 4; ALOX, arachidonate lipoxygenase; BH4, tetrahydrobiopterin; BSO, buthionine sulfoximine; CoQ, coenzyme Q; Cys, cysteine; Cys_2_, cystine; DHODH, dihydroorotate dehydrogenase; DMT1, divalent metal transporter 1; FPN, ferroportin; FSP1, ferroptosis suppressor protein 1; GCH1, GTP cyclohydrolase‐1; GCL, glutamate–cysteine ligase; Glu, glutamate; GPX4, glutathione peroxidase 4; GSH, glutathione; H₂O₂, hydrogen peroxide; LIP, labile iron pool; iNOS, inducible nitric oxide synthase; LPCAT3, lysophosphatidylcholine acyltransferase 3; MBOAT, membrane bound O‐acyltransferase; MUFA, monounsaturated fatty acid; LTF, lactotransferrin; NCOA4, nuclear receptor coactivator 4; PKCβII, protein kinase C; POR, cytochrome P450 oxidoreductase; PUFA, polyunsaturated fatty acid; SCD1, stearoyl‐CoA desaturase; Se, selenium; SFA, saturated fatty acid; STARD7, StAR‐related lipid transfer domain‐containing 7; STEAP3, six‐transmembrane epithelial antigens of the prostate 3; TF, transferrin; TFRC, transferrin receptor; TRP, transient receptor potential; VK, vitamin K.

Plasma membrane rupture represents the final phase in various forms of RCDs. In contrast to other RCDs that necessitate specific pore‐forming proteins for their execution, the occurrence of ferroptosis relies on distinct lipid‐centric mechanisms to disrupt plasma membrane integrity.^11^, ^12^ The peroxidation of PUFA‐PLs is a key event in ferroptosis, with oxidizable lipids, overloaded iron, and impaired antioxidant systems serving as critical prerequisites that drive this overwhelming peroxidation, ultimately resulting in plasma membrane rupture and ferroptotic cell death (Figure 1).

### 2.1 Ferroptosis prerequisites

#### 2.1.1 Oxidizable lipids

The preferential substrates in the cellular membrane, particularly PUFA‐PLs, are essential for lipid oxidation and ferroptosis onset. This is because PUFA tails within PLs contain more than one double bond and bis‐allylic moieties, which are highly vulnerable to oxidative damage and converted to PL hydroperoxides (PL‐PUFA‐OOHs).^13^ The incorporation of PUFA into PLs requires the action of lipid metabolism enzymes, specifically acyl‐CoA synthetase long‐chain family member 4 (ACSL4) and lysophosphatidylcholine acyltransferase 3 (LPCAT3).^14^ ACSL4 catalyzes the conversion of PUFA into PUFA‐CoAs, which can be integrated into PLs by LPCAT3.^15^ The activity of ACSL4 can be amplified by protein kinase C (PKCβII) through phosphorylation at Thr328 and dimerization, thus facilitating ferroptosis.^16^ Interestingly, recent research by Brent R. Stockwell's team has demonstrated that exogenous supplementation with PUFAs stimulates the biosynthesis of PLs with two PUFA tails (diacyl‐PUFA PLs; PL‐PUFA_2_s),^17^ rather than those with a single PUFA tail (PL‐PUFA_1_s), which were previously considered as the primary substrates for lipid peroxidation. The formation of PL‐PUFA_2_s facilitates mitochondrial reactive oxygen species (ROS) production and lipid peroxidation, thus inducing ferroptosis in multiple cancer cell lines.^17^ In addition to PUFA‐PLs, polyunsaturated ether phospholipids (PUFA‐ePLs), synthesized by peroxisomes, may also serve as substrates for lipid peroxidation and ferroptosis induction in certain cell lines.^18^, ^19^ Moreover, Ca^2+^‐independent phospholipase A2β (iPLA2β) can cleave oxidized PUFA residues from PLs to detoxicate lipid peroxidation and ferroptosis mediated by TP53 and ROS stress.^20^

In contrast, monounsaturated fatty acids (MUFAs) are protective against ferroptosis by competitively inhibiting the biosynthesis of PL‐PUFA.^17^ Biologically, endogenous MUFAs are converted by saturated fatty acids (SFAs) through stearoyl‐CoA desaturase, and ACSL3 catalyzes MUFAs into their corresponding MUFA‐CoAs.^21^, ^22^ Subsequently, members of membrane bound O‐acyltransferase (MBOAT) family, including MBOAT1 and MBOAT2, mediate the incorporation of MUFA‐CoA into PLs, thereby competitively reducing the levels of PLs with PUFA tails and suppressing ferroptosis.^23^ Exogenous supplementation with MUFAs, such as oleic acid, can hamper the interaction between PUFA and PL‐PUFA_1s_ to reduce PL‐PUFA_2_s content, particularly when co‐treated with PUFAs like docosahexaenoic acid.^17^ This process contributes to the ferroptosis‐inhibitory role of MUFAs. In addition to MUFAs, the enzyme LPCAT1‐mediated incorporation of both endogenous and exogenous SFAs into PLs counteracts the levels of PUFA‐PLs in the membrane, thereby conferring resistance to ferroptosis.^24^ In summary, the interplay between various lipids and associated lipid‐modifying enzymes is critical for regulating lipid peroxide production and determining vulnerability to ferroptosis.

#### 2.1.2 Overloaded iron

In line with its name, iron is a primary driving force behind ferroptosis through both nonenzymatic and enzymatic processes. In biological systems, iron mainly varies between ferrous (Fe^2+^) and ferric (Fe^3+^) redox states.^25^ Notably, the reaction between labile Fe^2+^ and hydrogen peroxide (H₂O₂) results in the formation of Fe^3+^ and hydroxyl radicals (OH•), a highly mobile and toxic form of ROS, and this process is commonly known as the Fenton reaction.^26^ Hydroxyl radicals produced by Fenton reaction can attack PUFA‐PLs to initiate and propagate nonenzymatic lipid peroxidation and ferroptosis.^27^ Moreover, iron and its derivatives serve as catalytic centers for various enzymes, such as arachidonate lipoxygenases (ALOXs) and cytochrome P450 (CYP450) oxidoreductase (POR), which are involved in the generation of lipid hydroperoxides.^27^ Hence, manipulating labile iron levels can regulate cellular sensitivity to ferroptosis by a series of iron metabolism processes, including iron import, storage, utilization, and export.

Under physiological conditions, Fe^3+^‐bound transferrin (TF) is recognized and internalized via the membrane protein transferrin receptor (TFRC)‐mediated endocytosis.^28^ Subsequently, Fe^3+^ can be reduced to Fe^2+^ by six‐transmembrane epithelial antigens of the prostate 3 within endosomes, and transported to the cytoplasm via divalent metal transporter 1 (DMT1/SLC11A2).^28^ The released ferrous iron contributes to the formation of labile iron pool (LIP), which initiates both nonenzymatic and enzymatic lipid peroxidation. Interfering with this endocytosis process by genetic inhibition of TFRC indeed decreases Fe^2+^ levels and relieves ferroptosis.^29^ Similarly, lactotransferrin is also implicated in the iron import and functions as a ferroptosis‐promoting factor.^30^ The excess Fe^2+^ can bind to ferritin for iron storage.^31^ Ferritin consists of ferritin heavy chain 1 (FTH1) and ferritin light chain (FTL), with only FTH1 possessing ferroxidase activity, which converts Fe^2+^ into nontoxic Fe^3+^ for storage.^32^ Consequently, inhibition of ferritin expression, especially FTH1, increases labile iron levels and promotes ferroptosis.^33^, ^34^ In response to intracellular iron demand, ferritin can be selectively degraded by nuclear receptor coactivator 4 (NCOA4)‐mediated ferritinophagy to increase iron availability, which dictates ferroptosis sensitivity.^35^, ^36^, ^37^ Additionally, excess Fe^2+^ can be exported by ferroportin (FPN/SLC40A1) in the cellular membrane. Therefore, it is not surprising that suppression of FPN expression can lead to increased cellular iron abundance and induce a proferroptotic state.^38^, ^39^ Together, the regulation of iron content through various factors controls cellular vulnerability to ferroptosis.

#### 2.1.3 Compromised antioxidant systems

Ferroptosis is antagonized by two main antioxidant systems, and impairment of these antioxidant mechanisms can induce or sensitize cells to ferroptosis. The first system involves glutathione (GSH) peroxidase 4 (GPX4), the only known enzyme that directly reduces membrane PL peroxides to alcohols to terminate lipid peroxidation.^40^ Notably, GPX4 is a selenoprotein, and selenium is essential for its expression and activity.^41^ Supplementation with selenium enhances both the transcription and protein synthesis of GPX4.^42^, ^43^, ^44^ In contrast, treatment with statins disrupts the translation of selenoprotein, particularly GPX4, leading to increased levels of cellular lipid peroxidation and heightened susceptibility to ferroptosis.^45^ GPX4 detoxifies PL peroxides dependent on its active site, namely selenocysteine. Small molecule inhibitors, such as RSL3, can react with the selenocysteine of GPX4, resulting in direct inactivation of GPX4 and potent induction of ferroptosis.^46^ Genetic ablation of GPX4 or enhanced degradation of GPX4 by pharmacological compounds (e.g., N6F11 and FIN56) also triggers an increase in lipid peroxides and consequent ferroptotic death.^47^, ^48^, ^49^ Furthermore, GPX4 exists in three isoforms: mitochondrial, cytosolic, and nuclear GPX4.^4^ Although mitochondrial GPX4 may contribute to inhibiting mitochondrial lipid peroxidation and ferroptosis, cytosolic GPX4 is generally regarded as the most crucial isoform for preventing ferroptosis.^50^ Increasing evidence suggests that cationic residues in cytosolic GPX4 enable electrostatic interactions with the plasma membrane surface, catalyzing the reduction of PL‐PUFA‐OOHs via a charge‐driven substrate recognition mechanism, despite the absence of plasma membrane‐targeting signals in cytosolic GPX4.^51^ GPX4 utilizes GSH as a cofactor to detoxify PL hydroperoxide.^40^, ^47^ GSH is synthesized from glycine, cysteine and glutamate via the enzyme glutamate–cysteine ligase (GCL), with cysteine serving as the rate‐limiting factor.^52^ Cells mainly import cystine (the oxidized form of cysteine) via the system xc^−^, a cystine‐glutamate antiporter composed of solute carrier family 7 member 11 (SLC7A11) and solute carrier family 3 member 2 (SLC3A2).^53^ Once imported, cystine is immediately reduced to cysteine through an NADPH‐consuming reaction.^54^ Consequently, blocking system xc^−^ activity with pharmacological agents including erastin, sulfasalazine (SAS), and sorafenib could result in impaired cystine uptake, GSH depletion, indirect inactivation of GPX4, and ultimate ferroptosis induction.^2^, ^55^, ^56^ Similarly, culturing cells in cystine‐starved medium triggers the rapid loss of GSH and GPX4 inactivation, thus inducing ferroptosis.^57^ Direct inhibition of GSH synthesis using the GCL inhibitor buthionine sulfoximine (BSO) also inactivates GPX4 and triggers ferroptosis in certain cell lines.^40^ Together, system xc^−^‐mediated cystine import, GSH synthesis, and GPX4 activity constitute a robust ferroptosis protection system, and intervention in this pathway can lead to ferroptotic cell death.

Although the system xc^−^–GSH–GPX4 system is the center of ferroptosis surveillance, GPX4‐independent mechanisms have been identified to protect against ferroptosis. Other systems are controlled by enzymes including ferroptosis suppressor protein 1 (FSP1),^58^, ^59^ dihydroorotate dehydrogenase (DHODH),^50^ GTP cyclohydrolase‐1 (GCH1),^60^ and inducible nitric oxide synthase (iNOS).^61^ These enzymes generate metabolites with lipophilic radical‐trapping antioxidant (RTA) properties, thereby effectively interrupting PL peroxidation cascades. Thus, interfering with these antioxidant systems can promote the accumulation of lipid peroxides and ferroptosis. FSP1 is a NAD(P)H‐ubiquinone reductase that can reduce coenzyme Q (CoQ) and vitamin K to their corresponding hydroquinone (CoQH_2_ and VKH_2_), which function as potent RTAs and prevent lipid peroxidation.^58^, ^59^, ^62^ Notably, the plasma‐membrane localization of FSP1, mediated by the N‐terminal myristoylation, is essential for its ferroptosis‐suppressing activity.^58^ Dissociation of FSP1 from the membrane and its phase separation induced by the compound icFSP1, or direct inhibition of FSP1 enzyme activity via iFSP1, could significantly enhance ferroptosis.^59^, ^63^ In addition, DHODH, a mitochondrial enzyme involved in pyrimidine biosynthesis, suppresses mitochondrial lipid peroxidation by reducing CoQ to CoQH_2_, which coordinates with mitochondrial GPX4 to prevent ferroptosis within the mitochondria.^50^ Inhibition or inactivation of DHODH has been linked to the promotion of ferroptosis in cancer cells characterized by low mitochondrial GPX4 expression. Furthermore, CoQ biosynthesis relies on StAR‐related lipid transfer domain‐containing 7 (STARD7) within mitochondria.^64^ The transport of CoQ from mitochondria to the cytoplasm and plasm membrane requires the presence of cytosolic STARD7.^64^ Both processes are essential for CoQ‐dependent antioxidant defense against ferroptosis.^64^ Moreover, GCH1 is implicated in the biosynthesis of the antioxidant tetrahydrobiopterin (BH4), which selectively protects PL‐PUFA_2_s from oxidative damage, thus safeguarding cells from ferroptosis.^60^ Inhibition of GCH1 expression or the regeneration of BH4 favors a proferroptotic state in cancer cells.^65^, ^66^ In addition to CoQH_2_, VKH_2_, and BH4, nitric oxide (NO^•^) derived from iNOS acts as another GPX4‐independent ferroptosis resistance factor, probably through its interaction with 15‐lipoxygenase (15‐LOX) and lipid radicals generated by 15‐LOX.^61^ M2 macrophages express lower levels of iNOS and NO^•^ compared with M1 macrophages, rendering them more vulnerable to ferroptosis.^61^ Metabolites involved in cholesterol biosynthesis, such as 7‐dehydrocholesterol (7‐DHC), also display ferroptosis‐modulating activity.^67^, ^68^, ^69^ By reducing lipid peroxyl radicals, 7‐DHC neutralizes PL peroxidation in both the plasma membrane and mitochondria, thus alleviating ferroptosis.^67^, ^68^ Inhibition of 7‐DHC synthesis through the deletion of the upstream enzyme sterol‐C5‐desaturase significantly increases cellular sensitivity to ferroptosis.^67^, ^68^ Collectively, these endogenous RTAs and associated metabolic enzymes form a complex network for scavenging deleterious lipid hydroperoxides. Inhibition of RTA biosynthesis by genetic or pharmacological approaches sensitizes cells to ferroptosis across diverse contexts.

### 2.2 Ferroptosis execution

In most forms of RCD, the terminal events typically involve permeabilization and plasma membrane rupture.^70^ Ferroptosis is primarily executed through mechanisms centered on lipid peroxidation, which regulates plasma membrane integrity. Lipid peroxidation occurs in three phases including initiation, propagation, and termination.^26^ The initiation of PL peroxidation involves both nonenzymatic and enzymatic mechanisms, as described above. Once PL peroxidation is initiated and not promptly neutralized, an auto‐amplifying lipid peroxidation chain reaction will occur. During this propagation process, the phospholipid radical (PL•) reacts with molecular oxygen, leading to formation of the phospholipid peroxyl radical (PLOO•). This peroxyl radical subsequently interacts with a PUFA within the PL, generating a lipid peroxide (PLOOH) and another new PL•, which can initiate another radical chain reaction. This autooxidation process can be terminated by GPX4 and GPX4‐independent antioxidant systems. Upon proferroptotic stimuli, the accumulation of lipid peroxides seems to occur in a sequential manner across different subcellular locations. Recent studies show that lipid peroxidation first accumulates in the endoplasmic reticulum membrane, followed by further cumulation in the plasma membrane, both of which are crucial for the initiation of ferroptotic cell death.^71^, ^72^

The accumulation of lipid peroxides in the plasma membrane could increase plasma membrane tension, which subsequently activates mechanosensitive cation channels, including Piezo1 and transient receptor potential.^12^ The opening of these channels causes an influx of Ca^2+^ and Na^+^, alongside an efflux of K^+^. Meanwhile, the inactivation of Na^+^/K^+^‐ATPase cooperatively potentiates the imbalance in ion fluxes.^12^ The loss of ion homeostasis and subsequent osmotic changes across the membrane lead to cell rounding and plasma membrane breakdown.^12^ Blocking this osmotic process with high molecular weight polyethyleneglycol, an osmoprotectant, significantly delays cell swelling, plasma membrane damage, and ferroptotic cell death.^73^, ^74^ The formation of nanoscale pores in the membrane has been observed during ferroptosis.^73^, ^74^ The opening of nanopores facilitates Ca^2+^ and water influx, leading to osmotic swelling, plasma membrane breakdown, and ultimately ferroptotic cell death.^73^, ^74^ However, it remains unclear whether pore‐forming proteins, such as gasdermin family proteins—known for mediating membrane rupture during pyroptosis—contribute to pore formation and permeabilization in ferroptotic cells. Furthermore, Ca^2+^ influx during ferroptosis could activate the endosomal sorting complexes required for transport (ESCRT)‐III complex.^73^, ^75^ ESCRT‐III typically mediates plasma membrane repair in response to necroptosis or pyroptosis.^76^, ^77^ Genetic inhibition of ESCRT‐III has been shown to increase sensitivity to ferroptosis, suggesting its membrane repair role in ferroptosis. These findings indicate the existence of a complex interplay within cells between plasma membrane damage and repair, and ferroptosis appears to execute when the damage is overwhelming.

## 3 EPIGENETIC AND NONEPIGENETIC REGULATION IN FERROPTOSIS

**FIGURE 2:**
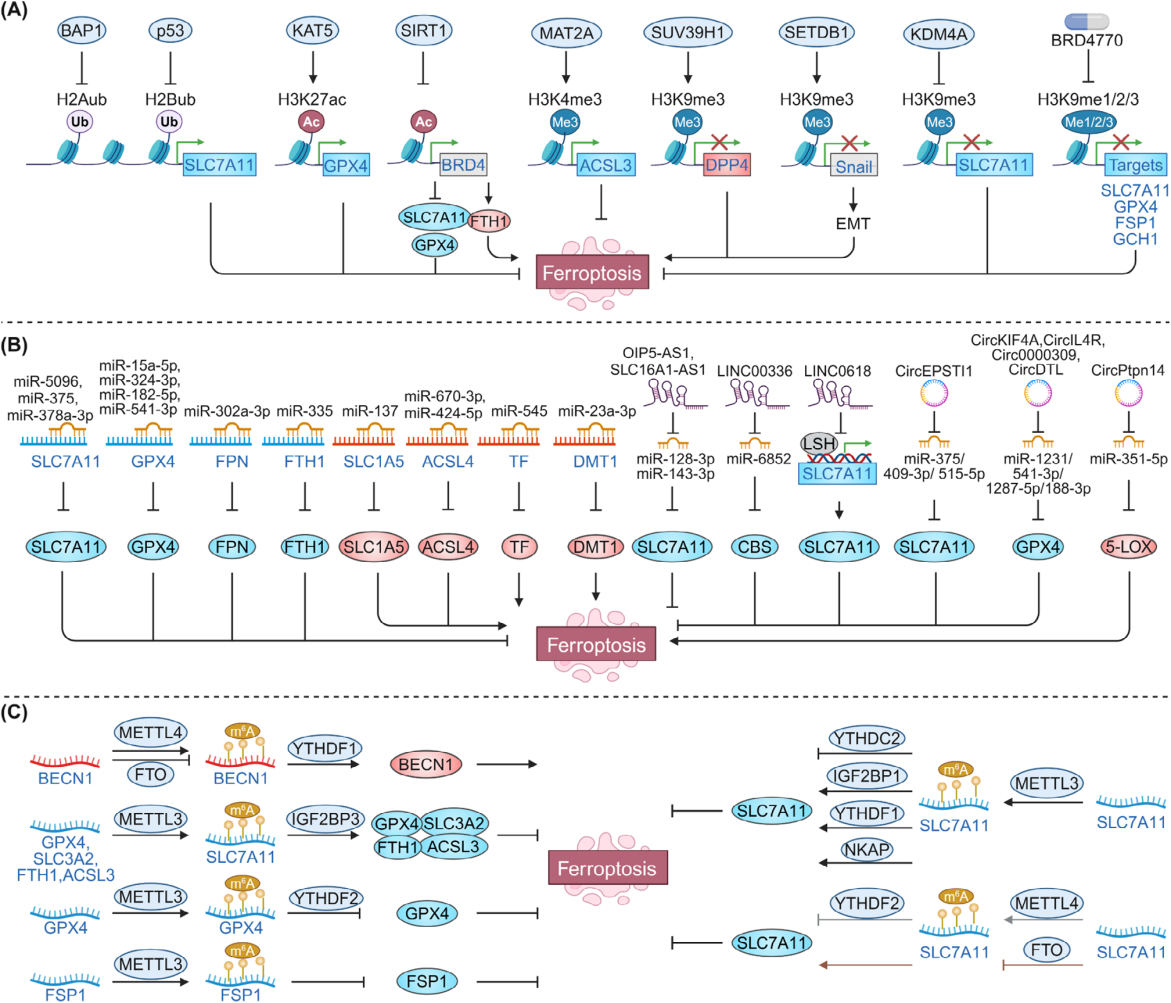
Epigenetic regulation in ferroptosis. (A) Posttranslational modifications of histones, such as acylation, methylation, and ubiquitination, regulate DNA accessibility and the expression of ferroptosis‐related genes, thereby modulating the cellular ferroptosis response. (B) miRNAs regulate ferroptosis by inhibiting mRNA translation or promoting mRNA degradation, while lncRNAs and circRNAs function as competing endogenous RNAs (ceRNAs), sponging miRNAs to modulate the expression of ferroptosis‐related genes such as SLC7A11 and GPX4. (C) m6A modifications regulate ferroptosis by altering the mRNA stability of key genes such as SLC7A11, GPX4, and FSP1 through the coordinated actions of methyltransferases, demethylases, and reader proteins. ACSL4, acyl‐CoA synthetase long‐chain family member 4; BAP1, BRCA1‐associated deubiquitinase 1; BECN1, beclin 1; CBS, cystathionine beta‐synthase; DMT1, divalent metal transporter 1; DPP4, dipeptidyl peptidase 4; FPN, ferroportin; FSP1, ferroptosis suppressor protein 1; FTH1, ferritin heavy chain 1; FTO, FTO alpha‐ketoglutarate dependent dioxygenase; GCH1, GTP cyclohydrolase‐1; GPX4, glutathione peroxidase 4; H2Aub, ubiquitination of histones H2A; H2Bub, ubiquitination of histones H2B; IGF2BP3, insulin‐like growth factor 2 mRNA binding protein 3; KAT5, lysine acetyltransferase 5; LSH, lymphoid‐specific helicase; NKAP, NF‐κB activating protein; m^6^A, N6‐methyladenosine; MAT2A, methionine adenosyltransferase 2A; METTL4, methyltransferase‐like 4; SIRT1, sirtuin 1; SLC1A5, solute carrier family 1 member 5; SLC3A2, solute carrier family 3 member 2; SLC7A11, solute carrier family 7 member 11; Snail, snail family transcriptional repressor 1; TF, transferrin; YTHDF1, YTH N6‐methyladenosine RNA binding protein F1.

**FIGURE 3:**
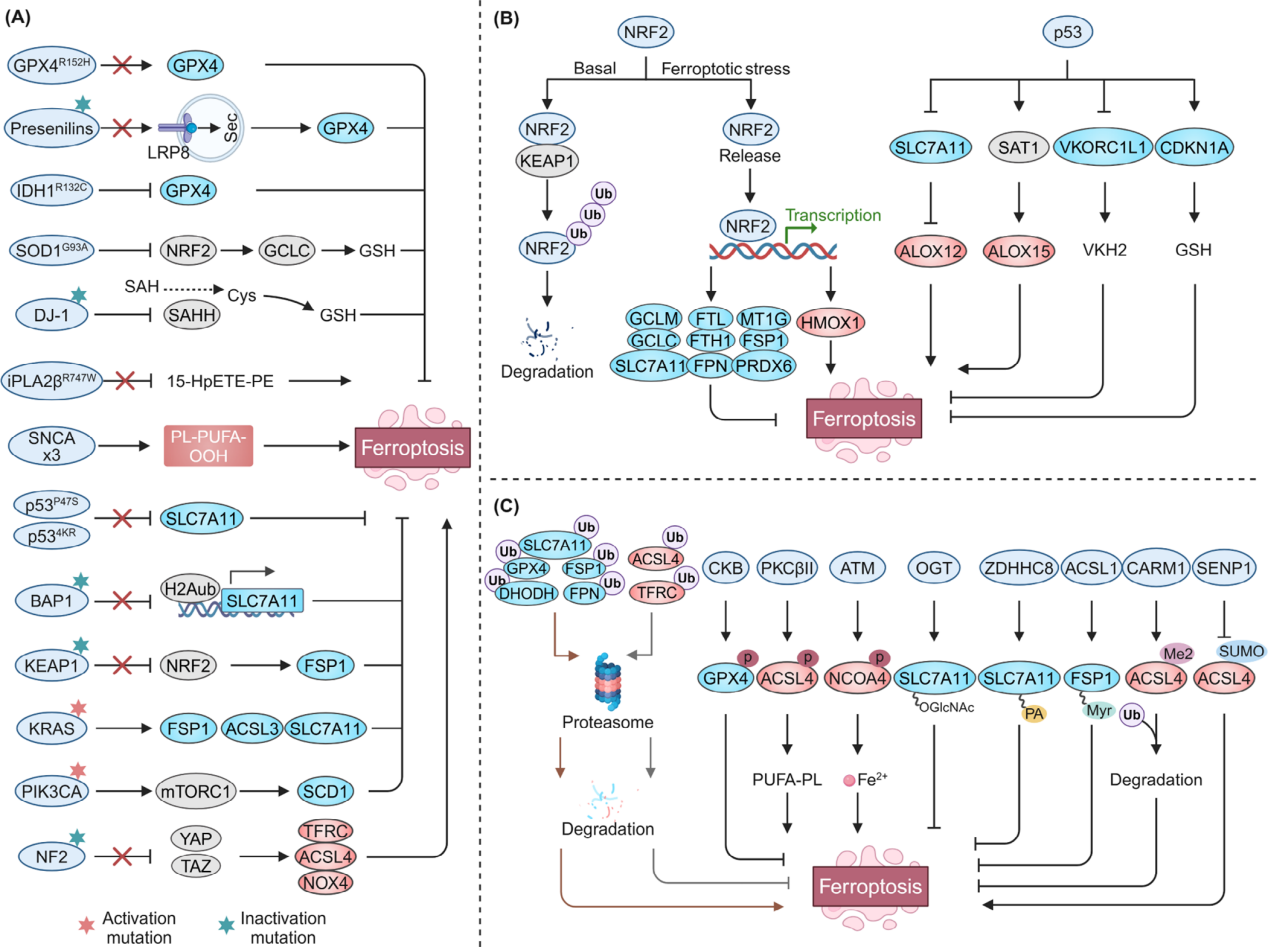
Nonepigenetic regulation in ferroptosis. (A) Genetic mutations in neurodegenerative diseases and cancers are key modulators of pathways influencing ferroptosis susceptibility as shown. (B) NRF2 transcriptionally regulates genes involved in GSH and GPX4 biosynthesis, iron metabolism, NADPH production, and FSP1, thereby modulating cellular susceptibility to ferroptosis. TP53 transcriptionally inhibits SLC7A11 and VKORC1L1 and upregulates SAT1, sensitizing cells to ferroptosis. However, under cystine deprivation, TP53 suppresses ferroptosis by promoting CDKN1A expression. (C) Core ferroptosis‐regulating proteins, including SLC7A11, GPX4, ACSL4, FSP1, and DHODH, can undergo multiple PTMs, such as ubiquitination, phosphorylation, acetylation, O‐GlcNAcylation, S‐palmitoylation, N‐myristoylation, methylation, and SUMOylation, thereby influencing ferroptosis sensitivity. ACSL4, acyl‐CoA synthetase long‐chain family member 4; ALOX, arachidonate lipoxygenase; BAP1, BRCA1‐associated deubiquitinase 1; CARM1, coactivator‐associated arginine methyltransferase 1; DHODH, dihydroorotate dehydrogenase; EGFR, epidermal growth factor receptor; FPN, ferroportin; FSP1, ferroptosis suppressor protein 1; FTH1, ferritin heavy chain 1; GCH1, GTP cyclohydrolase‐1; GPX4, glutathione peroxidase 4; GSH, glutathione; HMOX1, heme oxygenase 1; IDH1, isocitrate dehydrogenase 1; iPLA2β, phospholipase A2β; KEAP1, kelch‐like ECH‐associated protein 1; NF2, neurofibromin 2; NRF2, nuclear factor erythroid 2‐related factor 2; OGT, O‐linked N‐acetylglucosamine (GlcNAc) transferase; SAHH, s‐adenosylhomocysteine hydrolase; SAT1, spermidine/spermine N1‐acetyltransferase 1; SCD1, stearoyl‐CoA desaturase; SENP1, SUMO‐specific peptidase 1; SOD1, superoxide dismutase 1; TF, transferrin; TFRC, transferrin receptor; VKORC1L1, vitamin K epoxide reductase complex subunit 1‐like 1. GCLC, glutamate–cysteine ligase catalytic subunit; SNCA, synuclein α; PIK3CA, phosphatidylinositol‐4,5‐bisphosphate 3‐kinase catalytic subunit α; YAP, Yes1‐associated transcriptional regulator; TAZ, transcriptional coactivator with PDZ‐binding motif; NOX4, NADPH oxidase 4; CDKN1A, cyclin dependent kinase inhibitor 1A; CKB, creatine kinase B; ATM, ATM serine/threonine kinase; ZDHHC8, zinc finger DHHC‐type containing 8.

Ferroptosis response is governed by a complex network involving both epigenetic modifications (e.g., histone modifications, DNA methylation, ncRNAs, and m6A modification) and nonepigenetic modifications (e.g., genetic mutations, transcriptional regulation, and PTMs) (Figures 2 and 3). These modifications can influence its sensitivity or trigger ferroptosis by dynamically regulating the expression and activity of key ferroptosis‐related molecules, providing potential personalized targets for the development of ferroptosis‐based therapies.

### 3.1 Epigenetic regulation in ferroptosis

#### 3.1.1 Histone modifications

Histones are the fundamental protein components of chromatin. Histone octamers, consisting of two copies each of four histones (H2A, H2B, H3, and H4), are wrapped by DNA forming the basic repeating unit of chromatin known as the nucleosome. Various modifications, including acylation, methylation, and ubiquitination, have been identified on the amino termini or tails of histones, which regulate DNA accessibility and the expression of ferroptosis‐related genes (Figure 2A).

All histone core proteins are subject to ubiquitination, with H2A and H2B being the most frequently modified. SLC7A11 can be epigenetically activated through the ubiquitination of histones H2A (H2Aub) and H2B (H2Bub).^78^, ^79^, ^80^, ^81^ Tumor suppressors such as BRCA1 associated with deubiquitinase 1 (BAP1) and TP53 reduce the occupancy of H2Aub and H2Bub at the SLC7A11 promoter in a de‐ubiquitination‐dependent manner, suppressing its expression and inducing ferroptosis.^78^, ^79^ Recent studies have also shown that the histone H2A deubiquitinase MYSM1 is essential for hematopoietic stem cells function by protecting against ferroptosis. Mechanistically, MYSM1 deficiency reduces the translation rate of ferroptosis‐protective genes, thereby increasing the vulnerability of hematopoietic stem cells to ferroptosis and impairing their function.^82^

Histone acetylation typically serves as a positive regulatory modification, enhancing gene expression by reducing histone‐DNA interactions and loosening chromatin structure through the neutralization of histone's positive charge.^83^ Inhibition of lysine acetyltransferase 5 (KAT5), which reduces H3K27ac abundance at the GPX4 promoter, downregulates GPX4 and promotes ferroptosis in breast cancer cells.^84^ NAD^+^‐dependent histone deacetylases like SIRT1 and SIRT3 also induce ferroptosis by epigenetically inhibiting epithelial–mesenchymal transition in cancer cells.^85^, ^86^ Acetylation recognition by bromodomain‐containing (BRD) proteins, such as BRD4, further modulates ferroptosis sensitivity. The BRD4 inhibitor has been shown to elevate H3K4me3 and H3K27ac levels upstream of BRD4 by either inhibiting the histone methyltransferase G9a or enhancing histone deacetylase SIRT1 activity. This, in turn, disrupts BRD4's ability to recognize acetylation sites on histones at GPX4 and SLC7A11 genes, resulting in their downregulation and the subsequent induction of ferroptosis.^87^ Thus, BRD4 inhibitors could be used alone or in combination with immunotherapy to kill several BRD4‐proficient tumors by inducing ferroptosis.^87^, ^88^, ^89^ Furthermore, transcription factors like HIC1 and HNF4A compete with histone acetyltransferase KAT2B, impacting the transcriptional regulation of pro‐ and antiferroptosis genes and ferroptosis sensitivity.^90^

Histone methylation also plays a significant role, with different sites and levels of methylation conferring distinct functions. H3K4me3 and H3K9me3 are among the most studied methylation markers in ferroptosis. H3K4me3 typically promotes transcription, while H3K9me3 represses it.^91^ Methionine adenosyltransferase 2A (MAT2A) increases H3K4me3 occupancy at the ACSL3 promoter, enhancing ACSL3 expression and ferroptosis resistance.^92^ Histone methyltransferase SUV39H1 catalyzes H3K9me3 on the DPP4 promoter, thereby repressing its expression and DPP4–NOX1 complex formation, ultimately inhibiting lipid peroxidation and ferroptosis in clear cell renal carcinoma. The histone methyltransferase SETDB1 has been shown to promote ferroptosis by enhancing E‐cadherin expression through increasing H3K9me3 levels on the Snail promoter.^93^ In contrast, lysine demethylases KDM4A reduce H3K9me3 occupancy at the SLC7A11 promoter, upregulating SLC7A11 and resisting ferroptosis.^81^ Additionally, BRD4770 has been reported to activate the expression of key ferroptosis‐regulatory genes, including FSP1, SLC7A11, GPX4, and GCH1, by inhibiting H3K9me1/2/3 modifications, thus conferring resistance to ferroptosis in vascular smooth muscle cells.^94^

#### 3.1.2 DNA methylation

DNA methylation involves the addition of a methyl group to cytosine residues within DNA, a process mediated by DNA methyltransferases.^95^ This modification is typically associated with gene silencing.^95^ Key ferroptosis defense genes, such as GPX4 and FSP1, can be silenced by DNA methylation, enhancing ferroptosis susceptibility.^96^, ^97^ For instance, glycine increases levels of the DNA methylation donor SAM, thereby promoting GPX4 methylation and inducing ferroptosis in rheumatoid arthritis.^96^ Hypermethylation of the FSP1 promoter underpins the dependence of acute lymphoblastic leukemia on the GSH system, increasing its vulnerability to ferroptosis,^97^ while DNA methylation silences lipid metabolism genes like ELOVL fatty acid elongase 5 and fatty acid desaturase 1, conferring resistance to ferroptosis in gastric cancers.^98^

#### 3.1.3 ncRNAs regulation

ncRNAs, accounting for over 90% of human genome‐derived RNAs, play critical roles in biological processes and disease.^99^ ncRNAs are classified into various categories primarily according to their size, each playing a crucial role in regulating ferroptosis (Figure 2B). Among these, microRNAs (miRNAs) regulate ferroptosis by inhibiting messenger RNAs (mRNAs) translation or promoting mRNA degradation.^100^ Specific miRNAs, such as miR‐5096, miR‐375, and miR‐378a‐3p, induce ferroptosis through the downregulation of SLC7A11.^101^, ^102^, ^103^ Similarly, miRNAs including miR‐15a‐5p, miR‐324‐3p, miR‐182‐5p, and miR‐541‐3p facilitate ferroptosis by suppressing GPX4.^102^, ^104^, ^105^, ^106^ Conversely, miR‐137 inhibits ferroptosis by downregulating the glutamine transporter SLC1A5,^107^ while miR‐670‐3p and miR‐424‐5p reduce ferroptosis by targeting the lipid metabolism gene ACSL4.^108^, ^109^ Moreover, miRNAs act as versatile modulators of ferroptosis by influencing various iron metabolism processes. For example, miR‐302a‐3p and miR‐335 inhibit FPN expression and enhance FTH1 degradation, thereby increasing cellular iron levels and promoting ferroptosis.^110^, ^111^ In contrast, miR‐545 and miR‐23a‐3p target TF and DMT1 to prevent iron accumulation, thus suppressing ferroptosis.^112^, ^113^

LncRNAs also regulate ferroptosis, often functioning as competing endogenous RNAs (ceRNAs).^99^ By competitively binding to miRNAs, lncRNAs modulate the availability of miRNAs to interact with their target mRNAs, thereby influencing the expression of key genes involved in ferroptosis. For example, lncRNA OIP5‐AS1 and lncRNA SLC16A1‐AS1 upregulate SLC7A11 and inhibit ferroptosis by targeting miR‐128‐3p and miR‐143‐3p.^114^, ^115^ LncRNA LINC00336 promotes resistance to ferroptosis by upregulating cystathionine beta‐synthase and activating the transsulfuration pathway through competing with miR‐6852.^116^ Additionally, lncRNAs interact with proteins to regulate gene expression and ferroptosis susceptibility.^117^, ^118^, ^119^ For example, nuclear lncRNA LINC0618 interacts with lymphoid‐specific helicase, reducing its binding to the SLC7A11 promoter, thereby inhibiting SLC7A11 transcription and promoting ferroptosis.^120^ Similarly, cytosolic lncRNA LINC00472/P53RRA binds to G3BP stress granule assembly factor 1 (G3BP1), displacing TP53 and retaining it in the nucleus, which in turn promotes ferroptosis by affecting the transcription of multiple metabolic genes.^121^

Notably, circular RNAs (circRNAs), a subclass of lncRNAs generated through back‐splicing of pre‐mRNA, also act as ceRNAs in regulating ferroptosis sensitivity. CircEPSTI1 promotes SLC7A11 expression and inhibits ferroptosis by sponging several miRNAs, including miR‐375, miR‐409‐3p, and miR‐515‐5p.^122^ CircKIF4A, circIL4R, circDTL, and circ0000309 protect against ferroptosis by enhancing GPX4 expression via competitive binding to miR‐1231, miR‐541‐3p, miR‐1287‐5p, and miR‐188‐3p, respectively.^105^, ^123^, ^124^, ^125^ Conversely, circPtpn14 promotes ferroptosis by targeting miR‐351‐5p, which has been reported to inhibit the expression of 5‐LOX.^126^

#### 3.1.4 m6A modification

m6A is the most prevalent internal modification in eukaryotic mRNA and refers to a methylation that takes place at the N6 site of adenosine.^127^ This process is reversible, with methyltransferases (writers) adding the modification and demethylases (erasers) removing it, while reader proteins recognize the modified mRNA.^127^ m6A modifications regulate ferroptosis by altering the mRNA stability of ferroptosis‐related genes (Figure 2C). Elevated levels of m6A modification have been observed during ferroptosis in hepatic stellate cells, attributed to increased expression of methyltransferase‐like 4 (METTL4) and decreased expression of the demethylase FTO.^128^ Inhibiting this elevated m6A modification can suppress the stability of BECN1 mRNA mediated by the m6A reader protein YTH m6A RNA binding protein F1 (YTHDF1), thereby defending against ferroptosis.^128^ Consistently, demethylase FTO reduces SLC7A11 expression through m6A demethylation, sensitizing cells to ferroptosis.^129^ Methyltransferases METTL14 and METTL3 promote ferroptosis by accelerating the degradation of SLC7A11 mRNA through a YTHDF2 and YTHDC2‐dependent mechanism, respectively.^130^, ^131^ Interestingly, METTL3 can also stabilize and increase SLC7A11 mRNA levels, protecting against ferroptosis by facilitating the recognition of the m6A‐modified SLC7A11 motif by YTHDF1 and IGF2 mRNA‐binding protein 1.^132^, ^133^ Additionally, another m6A reader protein, NF‐κB activating protein, enhances the splicing of SLC7A11 mRNA in a METTL3‐dependent manner, further inhibiting ferroptosis.^134^ Several antiferroptotic genes, including GPX4, ACSL3, FTH1, and SLC3A2, are stabilized by METTL3‐mediated m6A methylation via IGF2BP3 recognition, contributing to desensitization to ferroptosis.^135^ METTL3 has also been implicated in promoting the methylation of FSP1 and GPX4 mRNAs, which inhibits their expression and increases vulnerability to ferroptosis.^136^, ^137^, ^138^

### 3.2 Nonepigenetic regulation in ferroptosis

#### 3.2.1 Genetic mutations

Ample evidence underscores the critical role of genetic mutations in the regulation of ferroptosis, laying the groundwork for understanding the involvement of ferroptosis in disease pathophysiology and identifying populations suitable for ferroptosis‐targeted therapies.^139^, ^140^, ^141^ Currently, research on the role of genetic mutations in ferroptosis regulation focuses on neurodegenerative diseases and cancers, which are closely associated with genetic mutations (Figure 3A).^139^, ^140^, ^141^, ^142^

Neurodegenerative disease‐causing mutations in proteins play an important role in modulating pathways that affect ferroptosis susceptibility, supporting the notion that ferroptosis serves as a mechanism in the development of neurodegenerative diseases. For example, the R152H missense mutation in GPX4 leads to partially reduced enzymatic activity and impaired ferroptosis resistance, which could be linked to the pathological phenotypes observed in spondylometaphyseal dysplasia patients carrying this mutation.^143^ Familial Alzheimer's disease and amyotrophic lateral sclerosis (ALS)‐associated mutations in presenilins and superoxide dismutase 1 confer ferroptosis vulnerability by inhibiting GPX4 expression through limiting LRP8‐mediated selenium uptake and impairing the NRF2 pathway and GSH synthesis, respectively.^144^, ^145^ Furthermore, Parkinson's disease‐related loss‐of‐function mutations in DJ‐1 (E64D, M26I, A104T, L166P) and iPLA2β (R747W) increase susceptibility to ferroptosis by suppressing s‐adenosylhomocysteine hydrolase‐mediated cysteine generation for GSH production and inhibiting the hydrolysis of 15‐HpETE from phosphatidylethanolamine (PE).^146^, ^147^ In addition, α‐synuclein triplication‐mediated increases in α‐synuclein levels confer vulnerability of neurons to ferroptosis by affecting ether‐linked phospholipid synthesis, contributing to familial Parkinson's disease pathology.^148^, ^149^

Moreover, loss‐of‐function mutations in tumor suppressor genes often confer resistance to ferroptosis, promoting tumorigenesis. For instance, acetylation‐deficient p53 mutants (p53 4KR: K117R, K161R, K162R, K98R) and the African‐specific S47 polymorphism (p53 P47S) contribute to ferroptosis resistance by impairing p53's ability to downregulate SLC7A11.^150^, ^151^ Similarly, loss‐of‐function mutations in BAP1 and kelch‐like ECH‐associated protein 1 (KEAP1) have also been reported to lose their ability to inhibit SLC7A11, thereby facilitating tumor growth.^78^, ^152^, ^153^ KEAP1 mutations have also been shown to upregulate FSP1 expression, leading to ferroptosis resistance in non‐small cell lung cancer cells.^154^ The activation of oncogenes can also confer resistance to ferroptosis. For example, oncogenic mutations in KRAS promote ferroptosis resistance by upregulating SLC7A11, ACSL3, and FSP1, while mutations in PIK3CA enhance mechanistic target of rapamycin kinase signaling, which also contributes to ferroptosis resistance.^155^, ^156^, ^157^, ^158^ Notably, in certain cases, mutations in tumour suppressors and oncogenes can confer vulnerability to ferroptosis in cancers. It was reported that inactivation of neurofibromin 2 renders cancer cells susceptible to ferroptosis through inhibiting YAP/TAZ signaling, whereas activation of epidermal growth factor receptor (EGFR) and isocitrate dehydrogenase 1 promote ferroptosis by inhibiting SLC7A11 and GPX4, respectively.^159^, ^160^, ^161^

#### 3.2.2 Transcriptional regulation

Transcriptional master regulators are critical in coordinating pathways that govern ferroptosis sensitivity. Numerous transcription factors, such as TP53,^162^, ^163^, ^164^ NRF2,^154^, ^157^, ^165^, ^166^, ^167^ NFE2L1,^159^, ^168^, ^169^ YAP1/TAZ,^159^ ATF3,^170^ HIF2α,^171^ ZEB1,^45^ STAT1,^172^ PPARα,^173^ and MYCN,^174^, ^175^, ^176^ can shape the ferroptosis threshold in cells by directly or indirectly modulating ferroptosis vulnerability‐governed genes or metabolites levels. Notably, the role of these transcription factors is context specific, as some transcription factors play significant roles in ferroptosis regulation in certain cell types but not in others.^4^ In this section, we focus on the complex roles of transcription factors NRF2 and TP53 in ferroptosis regulation (Figure 3B).

NRF2 is a major transcriptional activator of antioxidant defense mechanisms. Under basal conditions, NRF2 is bound by KEAP1 and undergoes proteasomal degradation, but during ferroptosis, it is released and translocated to the nucleus to promote the expression of target genes.^34^ Many of the genes involved in antiferroptotic pathway are targets of NRF2, such as genes involved in GSH biosynthesis (e.g., SLC7A11, GCLC, and GCLM), GPX4 synthesis (peroxiredoxin 6), iron regulation (e.g., FTH1/FTL and SLC40A1, and metallothionein 1G), and NADPH production (e.g., G6PD, PGD), as well as the key antiferroptosis factor FSP1.^153^, ^154^, ^177^, ^178^ NRF2 can positively regulate the transcription of these genes to resist ferroptosis. Notably, the role of NRF2 in resisting ferroptosis seems to be context‐dependent. In cells with high ferrous ion levels, it promotes ferroptosis by upregulating heme oxygenase 1 (HMOX1) expression.^179^

TP53, another crucial regulator, exhibits dual roles in ferroptosis regulation. As mentioned above, TP53 can transcriptionally and epigenetically repress SLC7A11 expression, sensitizing ferroptosis through an ALOX12‐dependent lipid peroxidation response.^79^, ^162^, ^180^, ^181^ Moreover, TP53 sensitizes ferroptosis by promoting polyamine catabolism through the upregulation of spermidine/spermine N1‐acetyltransferase 1 (SAT1) and inhibiting vitamin K synthesis via downregulation of vitamin K epoxide reductase complex subunit 1‐like 1.^163^, ^182^ However, TP53 can suppress ferroptosis under conditions of cystine deprivation by promoting the expression of CDKN1A, thereby conserving intracellular GSH.^164^

#### 3.2.3 PTMs

PTMs of ferroptosis‐related proteins are crucial in modulating ferroptosis susceptibility by influencing protein structure, activity, localization, and function (Figure 3C).^8^

Among these modifications, ubiquitination plays a pivotal role in protein degradation and stability via the proteasome system, directly impacting the levels of key proteins involved in ferroptosis.^183^, ^184^ Core ferroptosis proteins, such as SLC7A11,^185^ GPX4,^186^ ACSL4,^187^ FSP1,^188^ DHODH,^189^ and iron metabolism‐related proteins including TFRC and SLC40A1,^190^, ^191^, ^192^ can be directly labeled by ubiquitin to undergo ubiquitination‐proteasomal degradation, thereby affecting ferroptosis response. Moreover, linear ubiquitination, mediated by the HOIL‐interacting protein, has been shown to stabilize GPX4, thereby conferring protection against ferroptosis.^193^ Interestingly, ubiquitination's role extends beyond protein degradation to influencing protein localization, which further regulates ferroptosis. For instance, a recent study demonstrated that the E3 ubiquitin ligase TRIM21 mediates K63‐linked ubiquitination of FSP1 at Lys322 and Lys366, promoting its translocation to the plasma membrane, thus mitigating ferroptosis.^194^

Phosphorylation is another emerging mechanism regulating ferroptosis. Three key ferroptosis‐related proteins, GPX4, ACSL4, and NCOA4, are subject to phosphorylation, which modulates their ferroptotic function. Specifically, creatine kinase B‐induced phosphorylation of GPX4 at Ser104 inhibits ferroptosis by preventing its interaction with HSC70, thereby reducing autophagic degradation.^195^ Similarly, phosphorylation of ACSL4 at Thr328 by PKCBII enhances its dimerization and activity, driving ferroptotic cell death.^16^ Furthermore, the serine/threonine kinase ATM‐mediated phosphorylation of NCOA4 at Ser550 plays a crucial role in ferritinophagy and the ferroptosis induction.^37^

Acetylation, O‐GlcNAcylation, S‐palmitoylation, N‐myristoylation, methylation, and small ubiquitin‐like modifier (SUMO)ylation also contribute to ferroptosis regulation. For example, inhibition of acetylation in ALOX12 reduces ferroptosis susceptibility.^196^ O‐GlcNAc transferase‐mediated O‐GlcNAcylation and zinc finger DHHC‐type palmitoyltransferase 8 (ZDHHC8)‐mediated S‐palmitoylation of SLC7A11,^197^, ^198^ as well as ACSL1‐induced N‐myristoylation of FSP1,^58^, ^199^ enhance ferroptosis resistance. Conversely, coactivator‐associated arginine methyltransferase 1‐mediated methylation of ACSL4 reduces ferroptosis susceptibility, while inhibition of ACSL4 SUMOylation by SUMO‐specific peptidase 1 promotes ferroptosis.^200^, ^201^

Together, these findings underscore the multifaceted role of PTMs in the regulation of ferroptosis. The specific effects of PTMs on ferroptosis are likely determined by the substrate involved and the particular type and site of modification. A deeper understanding of the mechanisms through which PTMs regulate ferroptosis will provide insights for developing targeted therapies for ferroptosis‐related diseases.

## 4 THE ROLE OF FERROPTOSIS IN DISEASES

**FIGURE 4:**
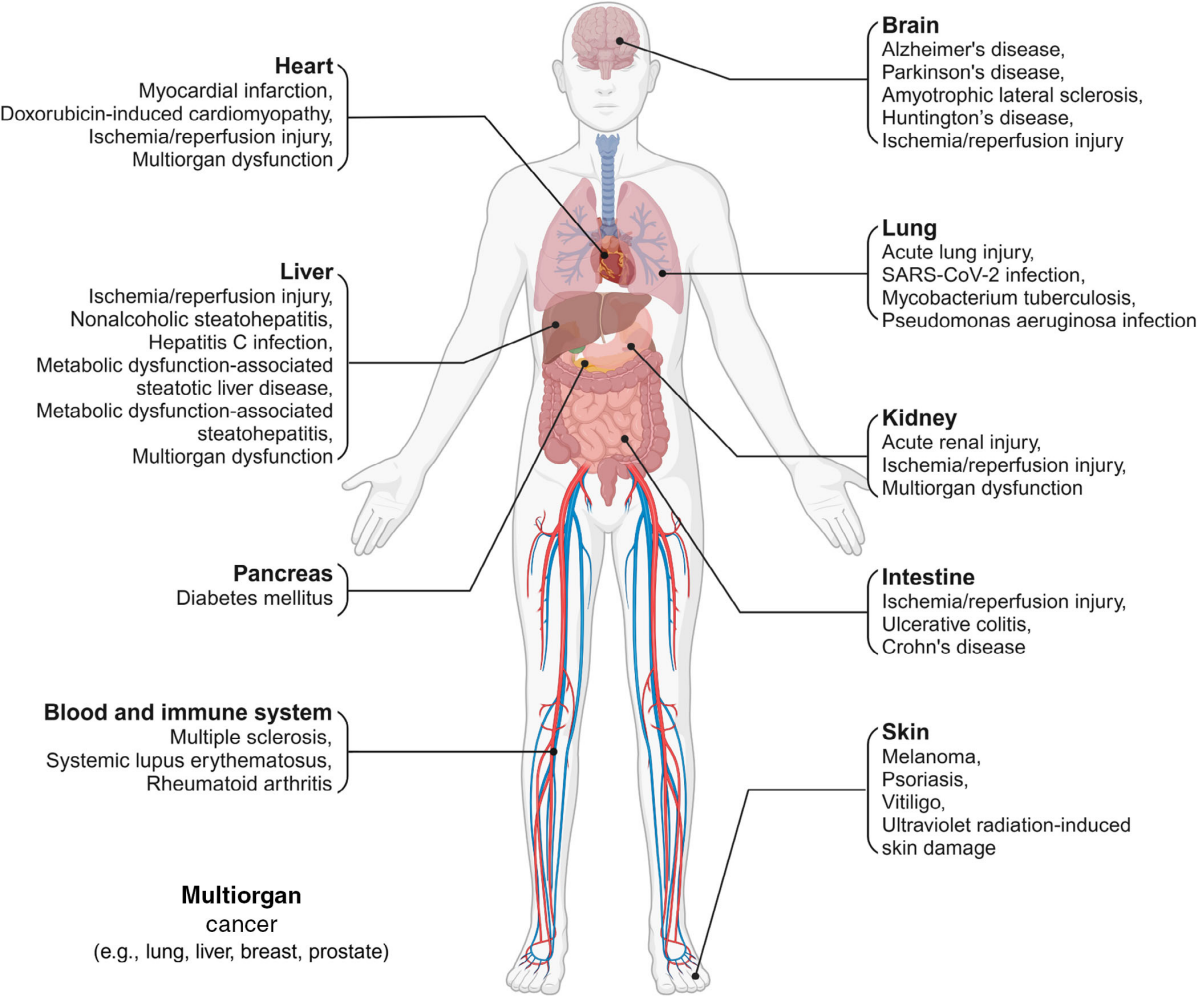
Role of ferroptosis in various diseases across different organs and tissues. Ferroptosis serves as an intrinsic tumor‐suppressive mechanism, with its evasion supporting tumorigenesis and progression. Additionally, ferroptosis activation is implicated in the pathogenesis of multiple neurodegenerative diseases, organ injuries, metabolic dysfunction‐associated steatotic liver disease, and dermatological conditions such as psoriasis, vitiligo, and UV‐induced skin damage. Notably, due to its complex interaction with the immune system, ferroptosis may exert dual effects, particularly in immune and infectious diseases.

In recent years, ferroptosis has been recognized as a physiological process vital for maintaining homeostasis, particularly in tumor suppression. Dysregulated ferroptosis is implicated in the pathogenesis of various diseases, including cancer, neurodegenerative diseases, organ injury, infectious diseases, autoimmune diseases, metabolic diseases, and skin diseases (Figure 4). Impaired system xc^−^–GPX4 pathways, iron overload and elevated oxidizable lipids contents are common key ferroptotic mechanisms mediating these diseases. Deciphering the specific cellular and molecular mechanisms triggering ferroptosis across different diseases will facilitate the development of disease‐specific ferroptosis‐targeted therapeutic approaches.

### 4.1 Cancer

The initial identification of ferroptosis in RAS‐mutant cancer cells through a cytotoxicity screening of compounds established a direct connection between ferroptosis and cancer pathophysiology.^2^ Since then, a growing body of evidence has elucidated the critical role of ferroptosis in tumor biology and its therapeutic potential.^202^, ^203^ Ferroptosis functions as an intrinsic defense mechanism against tumorigenesis and tumor progression.^162^ The inability to induce ferroptosis may promote tumor development. For instance, the retention of tumor‐suppressive function in p53^3KR^ mutant, which possesses ferroptosis‐promoting capabilities while lacking traditional abilities to promote cell‐cycle arrest, apoptosis, and senescence, alongside the loss of tumor‐suppressive function in p53^4KR^ mutant, which lacks ferroptosis regulatory activity, support the above viewpoint.^151^, ^162^ Besides, the reports of several tumor suppressor proteins function as ferroptosis promoters also provide evidence for this.^202^, ^204^

To counteract this intrinsic tumor suppressive mechanism, tumors have developed multiple mechanisms to evade ferroptosis, which supports their growth and metastasis.^5^, ^202^ These include inhibition of PUFA‐PLs synthesis and peroxidation, restriction of labile iron availability, and upregulation of cellular defense systems such as SLC7A11, GPX4, and FSP1, all of which enable cancer cells to bypass ferroptosis and continue proliferating.^5^ Of particular interest is the protective role of lymph fluid in metastasizing melanoma cells, where elevated levels of oleic acid and reduced free iron create an environment that shields these cells from ferroptosis, facilitating their metastasis.^205^

Despite these evasive mechanisms, certain cancer cells exhibit susceptibility to ferroptosis due to oncogene addiction and metabolic reprogramming, which provides a potential vulnerability for overcoming both intrinsic and acquired resistance to therapy.^5^, ^45^, ^160^, ^206^, ^207^ For example, EGFR‐mutant non‐small cell lung cancer cells, as well as de‐differentiated and persistent cancer cells are vulnerable to ferroptosis due to their dependence on cystine and metabolic rewiring or the acquisition of a mesenchymal state.^45^, ^160^, ^206^ Inducing ferroptosis has been functionally validated as an effective approach to suppress tumor growth across multiple cancer models in preclinical studies.^14^, ^34^, ^57^, ^208^

Additionally, ferroptosis not only directly modulates the fate of tumor cells but also plays a significant role in the tumor microenvironment to influence tumor development. Ferroptosis exhibits dual effects in antitumor immunity by directly modulating the fate and function of immune cells and indirectly inducing the release of multiple signals from ferroptotic cancer cells (e.g., DAMPs, MHC class I molecules, cytokines, and PTGS2).^202^ The complex interplay between ferroptosis and the tumor microenvironment highlights the significance of therapeutic time window for ferroptosis‐targeted therapy in cancer. On one hand, owing to the immunostimulatory effects of ferroptosis and its involvement in immunotherapy, ferroptosis induction appears to be a promising strategy for enhancing antitumor immunity and offers synergistic effects when combined with immunotherapy to kill well‐established tumors.^172^, ^209^, ^210^, ^211^ On the other hand, ferroptosis inhibition also could impede tumorigenesis, particularly in early‐stage tumors, due to the immune‐suppressive effect of ferroptosis in polymorphonuclear myeloid‐derived suppressor cells.^212^, ^213^ Thus, ferroptosis‐targeted therapy in cancer must consider the intricate nature of ferroptosis in the tumor microenvironment.

### 4.2 Neurodegenerative diseases

Neurodegenerative diseases, such as Alzheimer's disease, Parkinson's disease, ALS, and Huntington's disease, are characterized by progressive neuronal death and neurological dysfunction.^214^ Although the exact pathogenesis of neurodegenerative diseases remains unclear, a common pathophysiological hallmark, including iron accumulation and lipid peroxidation within affected regions, suggests that ferroptosis, an iron‐dependent form of cell death, plays a significant role in neuronal degeneration in these disorders.^27^, ^140^, ^215^, ^216^, ^217^, ^218^ Furthermore, glutamate excitotoxicity, a key contributor to neurodegenerative diseases, may also involve ferroptosis by inhibiting system xc^−^ and triggering STING‐dependent autophagic degradation of GPX4.^2^, ^219^, ^220^ Several pathogenic genes and proteins implicated in neurodegenerative diseases, such as DJ‐1 and PLA2G6 in Parkinson's disease and β‐amyloid and tau in Alzheimer's disease, have been associated with ferroptosis.^146^, ^147^, ^221^, ^222^

Experimental models have demonstrated that conditional genetic deletion of GPX4, a key ferroptosis regulator, leads to neurodegenerative phenotypes, including cognitive impairment and motor neuron death.^145^, ^223^ Ferroptosis inhibitors, lipophilic antioxidants, and iron chelators have shown promise in ameliorating neurodegeneration in ALS, Alzheimer's disease, and Parkinson's disease.^177^, ^223^, ^224^, ^225^ Indeed, several approved drugs for neurodegenerative diseases, such as idebenone for Alzheimer's disease, and edaravone for ALS, have been proven to alleviate neurodegeneration by inhibiting ferroptosis.^226^, ^227^, ^228^ Additionally, Copper(II)‐diacetylbis(N4‐methylthiosemicarbazone) (CuATSM), a novel drug currently undergoing clinical trials for patients with neurodegenerative diseases, has also been reported to possess antiferroptotic properties.^229^ All of these findings suggest that ferroptosis is a contributing factor in neurodegenerative diseases, and its inhibition may serve as a promising therapeutic strategy for these conditions.

### 4.3 Organ injury

Ferroptosis has been identified as a key driver of tissue injury across multiple organs, with varying susceptibility across different organs and cell types.^7^ Transgenic studies in mice have shown that proximal renal tubule cells are particularly sensitive to ferroptosis outside of the brain, as evidenced by the spontaneous development of acute renal failure in tamoxifen‐induced Gpx4 knockout mice.^47^ Ischemia/reperfusion‐induced organ damage, which underlies multiple devastating diseases, including myocardial infarction^230^ and stroke,^42^, ^231^ as well as injuries in other organs such as the liver,^47^ kidney,^232^ lung,^233^ and intestine,^234^ has been partially identified as a consequence of ferroptotic cell death. Inhibition of ferroptosis has consistently alleviated ischemia/reperfusion‐induced damage in various preclinical models. Furthermore, suppressing ferroptosis presents a promising therapeutic approach for improving outcomes in solid organ transplantation, where ischemia/reperfusion injury is an inevitable complication during implantation.^235^, ^236^

Beyond ischemia/reperfusion injury, ferroptosis has also been observed in other modes of organ injury, including doxorubicin (DOX)‐induced cardiomyopathy,^230^ rhabdomyolysis‐induced kidney injury,^237^ and environmental pollutants‐associated liver and lung injuries.^238^, ^239^ In critically ill patients, features of ferroptosis, including elevated malondialdehyde (MDA) and catalytic iron levels, have been observed in cases of multiorgan dysfunction, suggesting that ferroptosis inhibition may offer therapeutic benefits in these contexts.^240^

### 4.4 Infectious diseases

Ferroptosis has been reported to be activated during several infectious diseases and is associated with the burden of infections. Pseudomonas aeruginosa triggers ferroptosis in bronchial epithelial cells by secreting ALOX15, which catalyzes the oxidation of host arachidonic acid‐phosphatidylethanolamine (AA‐PE) into 15‐hydroperoxy‐AA‐PE (15‐HOO‐AA‐PE).^241^ Elevated levels of oxidized AA‐PE correlate with worse clinical outcomes, likely due to ALOX15‐mediated ferroptotic activity promoting persistent biofilm formation and compromising the bronchial epithelial barrier function.^241^ Similarly, Mycobacterium tuberculosis induces ferroptosis in macrophages, and ferroptosis inhibitors significantly reduce bacterial burden in infected mice.^242^ Supporting this, zebrafish Hmox1a protects against Mycobacterium marinum infection by limiting iron availability and reducing susceptibility to ferroptosis.^243^ In contrast, ferroptosis has been shown to inhibit hepatitis C virus (HCV) replication by altering the conformation of the HCV replicase complex through lipid peroxidation, with FADS2‐dependent fatty acid desaturation playing a key role.^244^ Additionally, ferroptotic characteristics, such as lipid alterations and upregulation of TFRC, have been observed in severe acute respiratory syndrome coronavirus 2 (SARS‐CoV‐2)‐infected hamsters, suggesting a potential link between SARS‐CoV‐2 infection and ferroptosis.^245^ However, the precise causal relationship between ferroptosis and these infections, as well as its role in subsequent inflammatory responses, requires further investigation.

### 4.5 Autoimmune diseases

Ferroptosis has been implicated in autoimmune diseases, such as multiple sclerosis, systemic lupus erythematosus (SLE), inflammatory bowel disease, and rheumatoid arthritis. Recent research identified STING‐dependent ferroptosis in neurons as a critical regulator in inflammation‐induced neurodegeneration, including multiple sclerosis.^220^ Moreover, ferroptotic neurons have been shown to enhance T‐cell activation by modulating T‐cell receptor signaling, thereby accelerating the progression of experimental autoimmune encephalitis, the murine model of multiple sclerosis.^246^ These findings suggest that ferroptosis plays a detrimental role in the development of multiple sclerosis. Similarly, in SLE, autoantibody‐ and interferon‐alpha‐mediated suppression of GPX4 induces ferroptosis in neutrophils, contributing to the immunopathogenesis of the disease. Inhibiting neutrophil ferroptosis has been shown to significantly reduce lupus severity in mice.^247^ Ferroptosis is also associated with the progression of SLE‐related conditions, such as lupus nephritis,^248^ likely due to the susceptibility of human proximal tubular cells to ferroptotic triggers present in lupus serum. Further research is needed to identify endogenous triggers of ferroptosis in SLE, which may illuminate the disease's pathological processes and provide novel therapeutic targets. In inflammatory bowel disease, ferroptosis is notably elevated in intestinal epithelial cells in both ulcerative colitis and Crohn's disease, accompanied by reduced GPX4 activity.^249^, ^250^ Additionally, Gpx4‐deficient mice fed with PUFAs develop enteritis resembling Crohn's disease, underscoring the role of ferroptosis in inflammatory bowel disease pathogenesis.^250^ In the context of rheumatoid arthritis, enhanced ferroptosis in chondrocytes and anti‐inflammatory macrophages correlates with disease progression and severity, while promoting ferroptosis in synovial fibroblasts has been shown to alleviate inflammation and improve symptoms.^251^, ^252^, ^253^ Therefore, targeting cell‐specific ferroptosis offers promising therapeutic strategies for autoimmune diseases.

### 4.6 Metabolic disease

Ferroptosis, as a consequence of dysregulated metabolism, has increasingly been linked to metabolic disorders, such as metabolic dysfunction‐associated steatotic liver disease (MASLD) and diabetes mellitus.^254^ In the liver, ferroptotic stress contributes to the onset and progression of MASLD.^255^ Initially, hepatic iron overload and lipid peroxidation induce ferroptosis, leading to the formation of lipid droplets and simple hepatic steatosis, characterized by lipid accumulation.^255^ Over time, excessive lipid buildup disrupts hepatic lipid metabolism, exacerbating ferroptosis‐mediated hepatocyte damage and triggering inflammatory responses. This can drive the progression from simple hepatic steatosis to metabolic dysfunction‐associated steatohepatitis (MASH), a more advanced stage of MASLD.^255^, ^256^, ^257^ In addition to hepatocyte ferroptosis, a recent study found that NCF1‐mediated ferroptosis in Kupffer cells worsens MASH progression.^258^ Inhibition of ferroptosis has been shown to significantly alleviate MASH and its progression toward fibrosis and hepatocellular carcinoma.^258^, ^259^, ^260^, ^261^, ^262^

In diabetes, ferroptosis‐associated pathways are activated in response to hyperglycemia.^263^, ^264^ Activated ferroptosis contributes to pancreatic β‐cell dysfunction and the development of various diabetic complications.^56^, ^263^, ^264^, ^265^, ^266^ However, a recent study revealed that the deficiency of CXCL16 in islet‐resident macrophages leads to excessive exposure to oxidized low‐density lipoprotein, which promotes ferroptosis in pathogenic CD8^+^ T cells, thereby inhibiting the progression of diabetes.^267^ These findings underscore the complexity of ferroptosis in metabolic diseases. Further research is required to elucidate the interactions between ferroptosis and tissue‐resident cells to better understand its role in metabolic diseases pathogenesis.

### 4.7 Skin diseases

Ferroptosis is also a key contributor to pathogenesis of various skin diseases.^9^ In psoriasis, ferroptosis triggers a cascade of inflammatory responses through lipid peroxidation in keratinocytes, contributing to the initiation and progression of psoriatic lesions.^9^ Studies have shown that ferroptosis inhibitors, such as ferrostatin‐1, can effectively alleviate inflammatory symptoms in psoriasis.^268^ Similarly, in vitiligo, ferroptosis is a key factor. The elevated iron content in melanocytes and impaired antioxidant defenses make these cells highly susceptible to ferroptosis, leading to skin depigmentation.^269^ Ferroptosis also exacerbates skin damage induced by ultraviolet (UV) radiation. UV‐exposed skin exhibits abnormal iron metabolism and increased lipid peroxidation, worsening skin damage. The application of ferroptosis inhibitors has been shown to effectively mitigate UV‐induced skin damage.^270^ In conclusion, ferroptosis plays a central role in the pathogenesis of various skin diseases. Drugs that modulate ferroptosis offer significant therapeutic potential, presenting promising avenues for treatment.

## 5 THERAPEUTIC APPROACHES TARGETING FERROPTOSIS

The role of ferroptosis in disease pathogenesis, both through its activation and inhibition, highlights lipid metabolism, iron homeostasis, and redox systems as key pathways for therapeutic intervention. Modulating these pathways offers a promising strategy for the treatment of ferroptosis‐related diseases. This section primarily summarizes clinical drugs that exhibit therapeutic potential by targeting key components of ferroptosis, providing a foundation for future clinical applications (Table 1).

**TABLE 1:** List of clinical trials associated with ferroptosis‐targeted drugs.

Agents	Mechanism and/or target	Effects	Experimental model	Outcomes	Indication	Trial phase and identifier number	References
**Targeting lipid metabolism pathway**							
Rosiglitazone	ACSL4 inhibitor	Inhibiting ferroptosis	Renal Gpx4−/− mice; I/R injury mice^14^	Reduces mortality associated with acute kidney injury; prevents I/R intestinal injury	Solid tumor malignancies	Phase II NCT04114136	^14^, ^234^
					Prostate cancer	Phase III NCT00182052	
					Ulcerative colitis	Phase II NCT00065065	
					HIV infection	Phase II NCT00367744	
					Sarcoma	Phase II NCT00004180	
					Alzheimer's disease	Phase I NCT00688207	
					MASH	Phase II NCT00492700	
					Kidney transplant	Phase II NCT00309309	
Baicalein	ALOX12/15 inhibitor; ACSL4 inhibitor	Inhibiting ferroptosis	Cisplatin and folic acid‐induced AKI in mice; CPT‐11‐induced delayed diarrhea in rat; heart I/R injury in rat	Alleviates renal inflammatory responses and AKI; alleviates colonic pathological injury and decreased inflammatory factor; improves myocardial I/R challenge‐induced ST segment elevation, coronary flow, left ventricular systolic pressure, infarct area, and pathological changes	Influenza	Phase II NCT03830684	^271^, ^272^, ^273^
Zileuton	ALOX5 selective inhibitor	Inhibiting ferroptosis	HT22 cells	Protects HT22 neuronal cells from glutamate oxidative toxicity in a ferroptosis‐dependent mechanism.	Chronic myelogenous leukemia	Phase I NCT02047149; Phase I NCT01130688	^274^
					Sickle cell disease	Phase I NCT01136941	
					Head and neck cancer; lung cancer	Phase II NCT00056004; Phase II NCT00070486	
					Tobacco use disorder	Phase II NCT02348203; Phase II NCT01021215	
					Acne vulgaris	Phase II NCT00098358	
NDGA	Pan‐LOX inhibitor	Inhibiting ferroptosis	Acute lymphoblastic leukemia cells	Blocks RSL3‐induced lipid peroxidation and cell death	Prostate cancer	Phase II NCT00678015	^275^
					High grade glioma	Phase I NCT02575794	
					Brain and central nervous System tumors	Phase I NCT00404248	
**Targeting iron homeostasis**							
DFO	Iron chelator	Inhibiting ferroptosis	MCD‐induced MASH in mice; hepatic I/R injury in mice; aged (15–18 months) C57 mice intraperitoneally injected with LPS; diabetic MCAO rat; MCAO rat	Attenuates the Severity of MASH; attenuates hepatic I/R injury and lipid peroxidation; improves inflammation and sickness behavior in aged mice treated with LPS; prevents vasoregression and microglia activation while improving AQP4 polarity as well as blood‐brain barrier permeability; reduce the area of cerebral infarction, improve the pathological structure of cerebral ischemia rats	Cardiomyopathy Hypotension, acute renal failure Ischemic stroke AKI Aneurysmal subarachnoid hemorrhage	Phase IV NCT00800761	^236^, ^266^, ^276^, ^278^
						Phase II NCT0087088	
						Phase II NCT00777140	
						Phase II NCT04633889	
						Phase II NCT04566991	
DFP	Iron chelator	Inhibiting ferroptosis	DSS‐induced ulcerative colitis in mice	Relieves the inflammation and impaired colon, and increase body weight	Acute myocardial infarction type 1	Phase I NCT05604131	^279^
					Neurodegeneration	Phase II NCT00907283	
					Stroke	Phase II NCT05111821	
DFX	Iron chelator	Inhibiting ferroptosis	Myocardial I/R injury in mice	Reduces myocardial injury and infarct size	Myelodysplasia	Phase II NCT03387475	^280^
					Sickle cell disease	Phase II NCT05392101	
DXZ	Iron chelator	Inhibiting ferroptosis	DOX‐ and I/R‐induced cardiomyopathy in mice	Prevents DOX‐induced cardiomyopathy and reduces the severity of cardiac I/R Injury.	During congenital heart surgery	Phase II NCT04997291	^230^
					Acute myeloid leukemia	Phase II NCT03589729	
**Targeting redox systems**							
SAS	SLC7A11 inhibitor	Promoting ferroptosis	H22‐luc hepatoma ascites mice	Reduces tumor burden	Glioma; glioblastoma; recurrent glioblastoma	Phase I NCT04205357	^281^
					Breast cancer; chronic pain due to malignancy	Phase II NCT03847311	
Sorafenib	SLC7A11 inhibitor	Promoting ferroptosis	MGC803 xenografts; HT‐1080 xenografts; 786‐O xenografts	Suppresses tumor growth	Hepatocellular carcinoma	Phase IV NCT01203787	^282^, ^283^, ^284^
					Acute myeloid leukemia	Phase III NCT01371981	
					Neuroblastoma	Phase II NCT02559778	
					Carcinoma, non‐small‐cell lung	Phase III NCT00449033	
BSO	Inhibition of GCL; GSH‐depleting	Promoting ferroptosis	BJ‐derived cell; HT‐1080 cells;	Induces selective lethality in BJ‐derived tumorigenic cells expressing oncogenic HRAS; inhibits HT‐1080 cells viability	Neuroblastoma	Phase I NCT00005835, Phase I NCT00002730	^40^, ^172^
Cisplatin	GSH‐depleting	Promoting ferroptosis	MKN‐45 xenografts; LLC xenografts	Suppresses tumor growth	NSCLC	Phase III NCT01656551	^285^, ^286^
					Bladder cancer	Phase III NCT04574960	
					Cervical cancer	Phase III NCT01561586	
					Cancer of pancreas	Phase II NCT03649321	
NAC	GSH synthesis regulator	Inhibiting ferroptosis	ICH in mice; polycystic ovary syndrome model in rats; diabetic nephropathy model in beagle; intermittent hypoxia‐induced myocardial injury in mice	Improves functional recovery at least 7 days following ICH in mice; attenuates gravid uterine and placental ferroptosis in a PCOS‐like rat model with fetal loss; ameliorates kidney injury in diabetic nephropathy; alleviates intermittent hypoxia‐related myocardial injury	Progressive MS	Phase II NCT05122559	^287^, ^288^, ^289^, ^290^
					Neurofibromatosis 1	Phase II NCT04481048	
					Skin disorder	Early phase 1 NCT05287724	
					Mitochondrial disease	Phase I NCT05241262	
					Diabetic neuropathies	Phase IV NCT04766450	
					Vascular cognitive impairment no dementia	Phase II NCT03306979	
					SLE	Phase II NCT00775476	
Mifepristone	Promoting GSH synthesis	Inhibiting ferroptosis	Acetaminophen induced liver injury in mice	Protects against APAP‐induced acute liver injury, evidenced by decreased ALT, AST level and histological recovery	Meningioma	Phase III NCT03015701	^291^
					Breast cancer	Phase III NCT05016349	
					NSCLC	Phase II NCT02642939	
					Prostate cancer	Phase II NCT00140478	
					Hepatitis C virus infection	Phase II NCT00255177	
					Endocrine disease; diabetes	Phase II NCT01419535	
WA	Alkylation of GPX4	Promoting ferroptosis	IMR‐32 xenografts	Suppresses the growth and relapse rate of neuroblastoma xenografts	Recurrent ovarian cancer	Phase I NCT05610735	^292^
Gemcitabine	Inhibiting GPX4	Promoting ferroptosis	A549 cells	Inhibits A549 cells proliferation	Pancreatic cancer	Phase II NCT06015659	^293^
					Biliary tract cancer	Phase II NCT05357196	
					Adult solid tumor	Phase I NCT05147272	
SeMet	GPX4 activators	Inhibiting ferroptosis	DOX‐induced acute cardiotoxicity in mice	Protects mice from DOX‐induced cardiotoxicity	Liver disease	Phase IV NCT01650181	^294^
					Precancerous/nonmalignant condition; prostate cancer	Phase III NCT00030901	
					Lung cancer	Phase II NCT00526890	
					Colorectal cancer	Phase II NCT00625183	
					ccRCC	Phase I NCT05363631	
Brequinar	DHODH inhibitor	Promoting ferroptosis	NCI‐H226 xenografts and TC494 lung cancer PDXs	Suppresses the tumor growth of GPX4^low^ xenografts	SARS‐CoV‐2 infection	Phase II NCT04575038	^50^
					Acute myeloid leukemia	Phase II NCT03760666	
Leflunomide	DHODH inhibitor	Promoting ferroptosis	SB‐driven hepatocarcinogenesis in mice	Constrains tumor mass and number, and achieves a much healthier liver and prolonged survival time	SARS‐CoV‐2 infection	Phase III NCT05007678	^189^
					Neuroendocrine tumors	Phase II NCT06540937	
					Brain and central nervous system tumors	Phase II NCT00003775	
					Idiopathic pulmonary Hemosiderosis	Phase II NCT05937191	
					Advanced pancreatic adenocarcinoma	Phase I NCT06454383	
Menaquinone‐4	RTA	Inhibiting ferroptosis	Hepatocyte‐specific Gpx4−/− mice; I/R injury in mice	Protects against related pathologic changes in liver; Protects against liver or kidney I/R injury in mice	Diabetes	Phase IV NCT00960973	^62^
					Osteoporosis	Phase IV NCT00548509	
					Hepatocellular carcinoma	Phase III NCT00165633	
Promethazine	RTA	Inhibiting ferroptosis	Cisplatin‐induced AKI and LPS/galactosamine‐induced liver injury in mice	Ameliorates AKI and increases the survival rate in mice; improves LPS/GalN‐induced acute liver injury	Pruritus	Phase IV NCT04805073	^295^
					Diabetic gastroparesis	Phase II NCT02130622	
Edaravone	RTA	Inhibiting ferroptosis	CSDS depression model in mice; permanent MCAO mice	Ameliorates depressive and anxiety‐like behaviors; alleviates cerebral ischemic injury in the mice with permanent MCAO	Acute ischemic stroke	Phase III NCT02430350	^296^, ^297^
					Nasopharyngeal carcinoma, brain necrosis	Phase II NCT01865201	
					Myocardial infarction	Phase IV NCT00265239	
					Cerebral infarction	Phase IV NCT00200356	
					ALS	Phase III NCT00415519	
CuATSM	RTA	Inhibiting ferroptosis	Mouse embryonic fibroblasts and hippocampal cells	Rescues embryonic fibroblasts and hippocampal cells from ferroptosis	ALS	Phase III NCT04082832	^298^

Abbreviations: ACSL4, acyl‐CoA synthetase long‐chain family member 4; AKI, acute kidney injury; ALOX12/15, arachidonate lipoxygenases 12/15; ALS, amyotrophic lateral sclerosis; ALT, alanine aminotransferase; APAP, acetaminophen; AST, aspartate aminotransferase; BSO, buthionine sulfoximine; ccRCC, clear cell renal cell carcinoma; CSDS, chronic social defeat stress; DFO, deferoxamine; DFP, deferiprone; DFX, deferasirox; DHODH, dihydroorotate dehydrogenase; DOX, doxorubicin; DSS, dextran sulfate sodium; DXZ, dexrazoxane; GCL, glutamate–cysteine ligase; Gpx4, glutathione peroxidase 4; GSH, glutathione; I/R, ischemia/reperfusion; ICH, intracranial hemorrhage; LLC, Lewis lung carcinoma; LPS, lipopolysaccharide; MASH, metabolic dysfunction‐associated steatohepatitis; MCAO, middle cerebral artery occlusion; MCD, methionine/choline‐deficient diet; MS, multiple sclerosis; NAC, N‐acetylcysteine; NCT, national clinical trial; NDGA, nordihydroguaiaretic acid; NSCLC, non‐small cell lung cancer; PCOS, polycystic ovary syndrome; PDXs, patient‐derived xenografts; RTA, radical‐trapping antioxidant; SARS‐CoV‐2, severe acute respiratory syndrome coronavirus 2; SAS, sulfasalazine; SB, sleeping beauty; SeMet, selenomethionine; SLC7A11, solute carrier family 7 member 11; SLE, systemic lupus erythematosus; WA, withaferin A.

### 5.1 Targeting lipid metabolic pathway

Since the incorporation of PUFA‐PLs into cell membranes is a prerequisite for ferroptosis, lipid metabolic pathways that modulate membrane lipid composition present promising therapeutic targets.^299^, ^300^ For example, thiazolidinediones, such as rosiglitazone, reduce mortality associated with acute kidney injury and prevent ischemia/reperfusion intestinal injury by selectively inhibiting ACSL4, an enzyme that facilitates the incorporation of PUFA‐PLs into membranes.^14^, ^234^ Similarly, baicalin, a natural flavonoid glycoside, has shown myocardial protection against ischemia/reperfusion by suppressing ACSL4‐mediated ferroptosis.^273^ However, clinical drugs targeting other key molecules involved in membrane lipid composition, such as LPCAT3, ACSL3, and MBOAT1/2, have yet to be identified. Interestingly, exogenous lipid supplementation, particularly a PUFA‐rich diet, may serve as an adjuvant therapy for ferroptosis‐related diseases, as it has been shown to delay tumor growth in colon cancer by enhancing acidosis‐driven ferroptosis. This effect is augmented by ferroptosis inducers such as SAS or erastin, and blocked by the inhibitor ferrostatin‐1.^301^

Additionally, peroxidation of PUFA‐PLs is a critical event in ferroptosis. ALOX enzymes, which catalyze PUFA oxidation, are important regulators of ferroptosis.^302^ The United States Food and Drug Administration (US FDA)‐approved drug Zileuton, an ALOX5 inhibitor, has shown neuroprotective effects by preventing ALOX5‐induced glutamate excitotoxicity and ferroptosis.^274^ Selective ALOX12/15 inhibitor baicalin has been found to effectively mitigate cisplatin‐induced acute kidney injury and CPT‐11‐induced gastrointestinal dysfunction by suppressing ALOX12/15‐dependent ferroptosis.^271^, ^272^ Pan‐LOX inhibitor nordihydroguaiaretic acid has also shown promise in inhibiting ferroptosis in acute lymphoblastic leukemia.^275^ However, its therapeutic effects and applications in improving ferroptosis‐induced diseases remain unclear, warranting further study.

### 5.2 Targeting iron homeostasis

Excess iron is a major driver of ferroptosis, and iron chelation therapies using agents like deferoxamine, deferiprone, and deferasirox can effectively alleviate diseases associated with iron overload, including neurodegeneration, organ injury, and MASH, by inhibiting ferroptosis.^236^, ^266^, ^276^, ^277^, ^278^, ^279^, ^280^, ^303^, ^304^ Dexrazoxane (DXZ), the only US FDA‐approved iron chelator for reducing DOX‐induced cardiotoxicity, also exhibits therapeutic effects related to ferroptosis by chelating mitochondrial iron.^230^ However, iron chelation poses risks of adverse effects such as anemia and renal toxicity, limiting its broader clinical application.^305^, ^306^, ^307^

Regulating iron homeostasis‐related molecules offers another potential therapeutic strategy for ferroptosis‐involved diseases. For example, baicalin, previously recognized for its antiferroptotic effects through the inhibition of ALOX12/15 and ACSL4, has also been shown to induce ferroptosis in bladder cancer cells by downregulating FTH1, inhibiting tumor growth.^308^ Clinical trials involving iron export regulators, such as the FPN inhibitor vamifeport and hepcidin antagonists PRS‐080, NOX‐H94, and LY2787106, which reduce intracellular iron levels by alleviating hepcidin‐mediated FPN suppression, have been reported. However, their potential to modulate ferroptosis in the management of ferroptosis‐related diseases has yet to be fully elucidated.^309^

### 5.3 Targeting redox systems

The maintenance of normal biological functions relies on the coordinated action of redox systems to preserve oxidative‐reductive homeostasis within the body. Dysregulation of key redox pathways, such as the system xc^−^–GSH–GPX4, FSP1–CoQ_10_–NAD(P)H, and DHODH systems, often leads to the onset of ferroptosis‐related diseases. Investigating clinically available drugs that target these redox systems is crucial for advancing the clinical translation of ferroptosis‐based therapies.

The system xc^−^‐GSH‐GPX4 pathway is a key guardian against ferroptosis. Small molecule inhibitors like erastin and RSL3 have been identified as research tools for targeting this pathway, while US FDA‐approved drugs such as antirheumatic SAS, antitumor kinase inhibitor sorafenib, and the muscle relaxant lanperisone have been shown to inhibit tumor growth by inducing ferroptosis through the inhibition of system xc^−^.^34^, ^55^, ^281^, ^282^, ^283^, ^284^, ^310^, ^311^, ^312^, ^313^, ^314^, ^315^ However, sorafenib's inability to induce ferroptosis in certain cancer cell lines highlights its limited clinical applicability for ferroptosis‐mediated tumor inhibition.^316^

In the context of GSH synthesis, as previously mentioned, BSO can induce ferroptosis in tumor cells by inhibiting GCL and depleting GSH.^40^, ^172^, ^317^ Despite its relative safety, BSO's clinical benefits for cancer patients remain limited.^318^ Identifying sensitive patient populations and exploring combination strategies may expand its clinical applications.^317^, ^319^, ^320^, ^321^ Cisplatin, a widely used antitumor drug, also induces ferroptosis by depleting GSH, adding another anticancer mechanism beyond apoptosis.^322^ Notably, cisplatin resistance is associated with ferroptosis resistance, and combining cisplatin with ferroptosis inducers may enhance efficacy in overcoming this resistance.^55^, ^285^, ^286^, ^323^ However, cisplatin‐induced ferroptosis is also implicated in chemotherapy‐related side effects, such as ovarian damage and acute kidney injury.^295^, ^324^ The antioxidant N‐acetylcysteine (NAC) has been shown to mitigate these side effects by inhibiting ferroptosis through modulation of cysteine metabolism.^287^, ^288^, ^289^, ^290^, ^324^, ^325^ This protective effect of NAC has also been observed in alleviating ferroptosis‐related acetaminophen (APAP) hepatotoxicity and neurodegeneration.^326^, ^327^ Furthermore, mifepristone has been shown to reduce APAP‐induced hepatotoxicity by promoting GSH synthesis.^291^

Inhibiting GPX4 directly triggers ferroptosis, with compounds like RSL3, ML162, and FIN56 showing preclinical potential. However, due to poor pharmacokinetics, their clinical applications remain limited. Natural compounds such as withaferin A and gemcitabine have shown tumor‐inhibiting effects by targeting GPX4, offering potential therapeutic avenues.^292^, ^293^, ^313^, ^328^ However, the direct inhibition of GPX4 requires caution, as GPX4 deficiency can cause severe side effects, such as acute kidney failure and embryonic lethality. Conversely, GPX4 activators, including selenium compounds like selenomethionine and selenocysteine‐containing peptides like Tat SelPep, have demonstrated the ability to alleviate DOX‐induced cardiomyopathy and promote stroke recovery by suppressing GPX4‐dependent ferroptosis.^42^, ^294^ The identification of other GPX4 activators with potent ferroptosis‐suppressing capabilities, such as 1d4 and natural compounds like curculigoside and puerarin, further expands the therapeutic potential of GPX4‐targeted treatments.^329^, ^330^, ^331^

The FSP1–CoQ_10_–NAD(P)H and DHODH systems function as parallel defense mechanisms against ferroptosis, making them promising therapeutic targets. Small molecules like iFSP1,^59^ NPD4928,^332^ and FSEN1^333^ selectively inhibit FSP1, sensitizing cancer cells to ferroptosis and providing a strategy to enhance therapies resistant to ferroptosis. In contrast, diphenylbutene derivative compounds 3f can inhibit ferroptosis by increasing FSP1 protein levels, offering protection against ischemic stroke.^334^ DHODH, which compensates for the loss of GPX4, also holds therapeutic potential for tumors with low GPX4 expression. Several clinical trials involving DHODH inhibitors, such as brequinar, leflunomide, and teriflunomide, have been reported.^50^, ^189^, ^335^ Combining DHODH inhibitors with AMPK activators, SAS, or oxaliplatin has shown synergistic tumor inhibition through ferroptosis induction.^50^, ^189^, ^336^

Free radicals play a crucial role in lipid peroxidation, driving ferroptosis through the formation and propagation of lipid radicals. RTAs can block these processes, with lipophilic RTAs such as ferrostatin‐1 and liproxstatin‐1 being potent specific ferroptosis inhibitors.^2^ Compared with ferrostatin‐1, liproxstatin‐1 demonstrates superior pharmacokinetics and efficacy in treating various ferroptosis‐related diseases in vivo, such as hepatic ischemia/reperfusion injury and acute renal failure.^47^ However, liproxstatin‐1's inhibition of CYP450 limits its clinical utility, as this inhibition may slow down drug metabolism and clearance, thus increasing the risk of adverse effects.^47^ Recent study suggests that sulfane sulfur species, particularly hydropersulfides (RSSH), may act as endogenous radical scavengers and could offer novel approaches to ferroptosis treatment.^337^, ^338^ Furthermore, vitamin K derivative hydroquinone, reduced by FSP1, exhibits RTA functionality and significant antiferroptotic effects.^62^ Additionally, diarylamine derivatives, such as phenothiazine, a key pharmaceutical core structure, have been identified as effective RTA inhibitors. Among them, the derivative drug promethazine demonstrates a stronger ability to protect kidney function compared with ferrostatin‐1, highlighting the potential therapeutic applications of diarylamine derivatives in treating ferroptosis‐related diseases.^295^, ^339^ The clinically approved RTA edaravone, used to treat cerebral ischemic injury and ALS, likely exerts its neuroprotective effects through ferroptosis suppression.^297^, ^340^, ^341^ CuATSM, a clinical candidate for treating Parkinson's disease and ALS, also suppresses ferroptosis via its RTA activity.^298^ These discoveries underscore the importance of RTAs in ferroptosis‐related therapies, offering new opportunities for clinical translation.

## 6 POTENTIAL CLINICAL MONITORING APPROACHES FOR FERROPTOSIS

Given the involvement of ferroptosis in disease pathogenesis and therapy, identifying effective clinical monitoring approaches is crucial. Several biomarkers of ferroptosis have been described (Table 2), including mitochondrial alterations (e.g., shrinkage, increased membrane density, and reduced cristae), elevated ferrous iron levels and iron metabolism, increased lipid peroxidation along with its byproducts (e.g., lipid ROS, MDA, and 4‐hydroxynonenal [4‐HNE]), and changes in ferroptosis‐related genes (e.g., CHAC1, PTGS2, SLC7A11, and ACSL4).^342^, ^343^, ^344^, ^345^ While elevated ferrous iron levels and lipid peroxidation are key biomarkers for ferroptosis, their transient nature and low expression levels pose challenges for direct detection in clinical settings.^342^, ^344^, ^345^ Biomarkers such as 4‐HNE, MDA, hyperoxidized peroxiredoxin 3, and TFRC show relative potential for clinical detection of ferroptosis due to their stability and detectability in tissue sections.^202^ However, these molecules indicate the ferroptotic response in an indirect and static manner. More potential clinical monitoring approaches for detecting ferroptosis directly and dynamically should be developed.

**TABLE 2:** Summary of ferroptosis biomarkers.

Name	Property	Alteration	Detection technique	Samples	Description	References
Lipid ROS	Oxidation product	Up	BODIPY 581/591 C11 probes	Cells	Detecting ROS formed from hydroperoxides, but not to hydroperoxides themselves	^2^, ^346^
4‐HNE	Oxidation product	Up	Antibody staining	Cells; ex vivo tissue	Aldehyde secondary products of lipid peroxidation	^347^, ^348^
MDA	Oxidation product	Up	TBARSs assay; antibody staining	Cells; ex vivo tissue	Aldehyde secondary products of lipid peroxidation	^349^, ^350^
TFRC	Protein	Increased plasma localization	3F3‐FMA antibody and additional anti‐TFRC antibodies	Cells; ex vivo tissue	Selectively staining ferroptotic cells in tissue sections	^351^
PRDX3	Protein	Hyperoxidized and translocated from mitochondria to plasma membranes	Antibody staining	Cells; ex vivo tissue	Identifying ferroptotic cells in tissue sections	^352^
PTGS2	mRNA	Up	qRT‐PCR assay	Cells; ex vivo tissue	Upregulated in specific contexts	^40^, ^347^
CHAC1	mRNA	Up	qRT‐PCR assay	Cells; ex vivo tissue	Primarily upregulated by system xc^−^ inhibitors, but not by other ferroptosis inducers	^56^
SLC7A11	Protein	Up	Antibody assay	Cells; ex vivo tissue	Upregulated in specific contexts	^311^
ACSL4	Protein	Up	Antibody assay	Cells; ex vivo tissue	Upregulated in specific contexts	^353^

Abbreviations: 4‐HNE, 4‐hydroxynonenal; ACSL4, acyl‐CoA synthetase long chain family member; CHAC1, ChaC glutathione‐specific gamma‐glutamylcyclotransferase 1; MDA, malondialdehyde; PRDX3, peroxiredoxin 3; PTGS2, prostaglandin‐endoperoxide synthase 2; ROS, reactive oxygen species; SLC7A11, solute carrier family 7 member 11; TBARSs, thiobarbituric acid reactive substances; TFRC, transferrin receptor; qRT‐PCR, quantitative real‐time PCR.

Imaging techniques are gaining attention for their ability to detect ferroptosis both spatially and temporally. Fluorescence‐ and probe‐based methods are regarded as key approaches in ferroptosis imaging. Three such techniques, BODIPY 581/591 C11, photochemical activation of membrane lipid peroxidation, and hydrogen peroxide fluorescent probes, have been developed to capture and visualize ferroptotic processes.^346^, ^354^, ^355^, ^356^ Among these, BODIPY 581/591 C11 has become a primary method for identifying ferroptosis by specifically detecting lipid ROS production. However, these techniques are largely restricted to tissue sections and cell cultures, limiting their use for dynamic, in vivo detection of ferroptotic activity in clinical settings. Similarly, mass spectrometry remains limited to the detection of lipid hydroperoxide levels in ex vivo tissue samples during ferroptosis.^61^ Notably, this technique offers detailed insights into lipid molecular mass, elemental composition, and chemical structure throughout the ferroptotic process, facilitating the identification of potential biomarkers for ferroptosis.^72^, ^357^ Moreover, positron emission tomography (PET) imaging with tracers like 18F‐TRX, 18F‐FSPG, and 68Ga‐NOTATf enables real‐time, noninvasive monitoring of ferroptosis in vivo, by measuring the intracellular LIP, system xc^−^ activity, and TF uptake, respectively.^358^, ^359^, ^360^, ^361^, ^362^ Notably, ongoing clinical trials using 18F‐FSPG for PET imaging in tumor patients represent a significant step forward in detecting system xc^−^ activity, potentially revolutionizing the clinical monitoring of ferroptosis.^361^, ^362^

Another emerging technique for noninvasive in vivo imaging of ferroptosis is magnetic resonance imaging (MRI), which excels at resolving soft tissue and anatomical structures. Recently, an artemisinin‐based probe (Art‐Gd) was developed for contrast‐enhanced MRI to detect ferroptosis, leveraging the radical formation reaction between labile Fe^2^⁺ and artemisinin. This reaction allows Art‐Gd to form complexes that enhance tissue retention and improve longitudinal relaxation time (T1) contrast, enabling real‐time MRI detection of ferroptosis in vivo.^363^ However, MRI's limitations in molecular‐scale imaging pose challenges, as there is a scarcity of contrast agents capable of detecting other ferroptosis‐related molecules such as lipid peroxides, GSH, and iron transporters. Further research is needed to overcome these limitations and enable the detection of multiple ferroptosis biomarkers, providing a more accurate and comprehensive assessment of this process.

## 7 OPPORTUNITIES AND CHALLENGES

Numerous preclinical studies suggest that targeting ferroptosis offers promising therapeutic opportunities for related conditions. However, our understanding of ferroptosis remains insufficient, with several unresolved issues potentially hindering its clinical translation.

Although overwhelming lipid oxidation and subsequent plasma membrane rupture are recognized as central events in ferroptosis, the intricate cellular biology underlying this process, including intracellular lipid transport between organelles, the propagation of lipid peroxidation reactions within cells, the degradation of specific lipid species during peroxidation, and the role of fragmented oxidative products in membrane rupture—remains to be fully elucidated.^4^ Additionally, much of the current understanding of ferroptosis stems from studies in cultured cancer cells. More research is needed to explore ferroptosis in cell lines from other diseases and normal tissues, as these may involve distinct mechanisms.^4^ A comprehensive understanding of the biological activities of ferroptosis is essential for advancing research in this field.

A host of molecules and metabolic products related to lipid and iron metabolism, as well as redox systems, have been identified as regulators of ferroptosis. However, further research is required to identify and quantify endogenous triggers and inhibitors of ferroptosis across various pathophysiological states. This will enhance our understanding of ferroptosis responses in different individuals, tissues, and conditions, elucidate the causal relationships between ferroptosis and diseases, and guide the development of targeted biomarkers and therapies, as well as the evaluation of their efficacy and side effects.^364^

In terms of clinical monitoring, assessing ferroptosis remains in its early stages. There is a need for more specific and less toxic detection reagents to enable precise, noninvasive real‐time in vivo monitoring of ferroptosis. Given the specific substances released from ferroptotic cells, developing detection methods based on blood, urine, and fecal samples represents a promising future direction. Additionally, identifying populations most likely to benefit from ferroptosis‐targeted therapies and achieving precise, ferroptosis‐specific treatments are key goals in ongoing ferroptosis research.

## AUTHOR CONTRIBUTIONS

Guangtong Deng designed the review. Qian Zhou and Yu Meng searched for literature and wrote the manuscript. Qian Zhou and Yu Meng drew the figures and tables. Jiayuan Le, Yuming Sun, Yating Dian, Lei Yao, Yixiao Xiong, Furong Zeng, and Xiang Chen and helped edit and revise the manuscript. Guangtong Deng, Qian Zhou, Y. L., and Furong Zeng provided funding support. All authors have read and approved the article and agree with publication in this journal.

## CONFLICT OF INTEREST STATEMENT

The authors have declared that no conflict of interest exists.

## ETHICS STATEMENT

Not applicable.

## Data Availability

Not applicable.
